# Supplementary material for: Quantification of spatial subclonal interactions enhancing the invasive phenotype of pediatric glioma
Source: Cell Rep. 2022 Aug 30;40(9):111283. doi: 10.1016/j.celrep.2022.111283 (PMC9449134; doi:10.1016/j.celrep.2022.111283)
Supplement: Document S1. Figures S1–S4 and Tables S1 and S2 [file mmc1.pdf]

Cell Reports, Volume *40*

Supplemental information

Quantification of spatial subclonal interactions enhancing the invasive phenotype of pediatric glioma

Haider Tari, Ketty Kessler, Nick Trahearn, Benjamin Werner, Maria Vinci, Chris Jones, and Andrea Sottoriva

Supplementary information

Origin Tumour	Condition	Doubling time (days)
SU-DIPG-VI	Bulk tumour	3.4
SU-DIPG-VI	VI-D10	4.2
SU-DIPG-VI	VI-E6	4.6
SU-DIPG-VI	50% VI-D10 + 50% VI-E6	4.4
SU-DIPG-VI	75% VI-D10 + 25% VI-E6	4.2
SU-DIPG-VI	25% VI-D10 + 75% VI-E6	4.3
HSJD-DIPG-007	Bulk tumour	2.2
HSJD-DIPG-007	007-F8	2.1
HSJD-DIPG-007	007-F10	1.9
HSJD-DIPG-007	50% 007-F8 + 50% 007-F10	2.0
HSJD-DIPG-007	75% 007-F8 + 25% 007-F10	2.1
HSJD-DIPG-007	90% 007-F8 + 10% 007-F10	2.1

Supplementary Table 1: Doubling time for SU-DIPG-VI and HSJD-DIPG-007 derived subclones in mono-culture and various co-culture. Doubling times derived from 2D in vitro growth assays, are not directly comparable to growth under invasion assays but highlight little effect of co-culture conditions on growth rates observed. SU-DIPG-VI clones and bulk tumour line growth rate (n=3). HSJD-DIPG-007 clones and bulk tumour line growth rate (n=1).
(Related to cell doubling times in STAR Methods)

Experiment ID	Condition	Number of images				Comment
		Day 0	Day 1	Day 2	Day 3	
1	100% VI-E6	3	3	3	3	In this assay VI-E6 was labelled with Venus
1	90% VI-E6 + 10% VI-D10	3	3	3	3	
1	70% VI-E6 + 30% VI-D10	7	6	6	9	
1	50% VI-E6 + 50% VI-D10	9	10	10	11	
1	40% VI-E6 + 60% VI-D10	4	8	8	8	
1	20% VI-E6 + 80% VI-D10	6	8	7	8	
2	100% VI-D10	4	4	4	4	In this assay VI-D10 was labelled with Venus
2	10% VI-E6 + 90% VI-D10	3	3	3	3	
2	50% VI-E6 + 50% VI-D10	3	2	2	2	
2	90% VI-E6 + 10% VI-D10	5	5	4	4	
3	100% VI-E6	3	2	2	1	In this assay VI-E6 was labelled with Venus
3	90% VI-E6 + 10% VI-D10	5	6	6	7	
3	50% VI-E6 + 50% VI-D10	5	6	6	6	
3	10% VI-E6 + 90% VI-D10	2	2	3	4	
4	100% 007-F8	4	4	4	4	In this assay 007-F8 was labelled with mCherry and 007-F10 was labelled with Venus
4	90% 007-F8 + 10% 007-F10	3	3	3	3	
4	75% 007-F8 + 25% 007-F10	6	6	6	6	
4	50% 007-F8 + 50% 007-F10	4	4	4	4	
4	100% 007-F10	4	4	4	4	

Supplementary Table 2: Summary of the number of timepoints taken per condition for invasion assays between SU-DIPG-VI and HSJD-DIPG-007 derived subclones. There are variations between number of images per condition between timepoints. This is due to out of focus images being left out from analysis. For SU-DIPG-VI the mCherry labelling was not detectably expressed. This is a known issue with RFP-like labelling and as such for clones from this cell line only the green channel was used to track a subpopulation. In all assays both subclones were labelled with either mCherry (red) or Venus (green)

Experiment ID 1 = Counts of images for assays where VI-E6 is labelled with Venus and tracked (Assay 1)**. Experiment ID 2 =** Counts of images for assays where VI-D10 is labelled with Venus and tracked. **Experiment ID 3 =** Counts of images for assays where VI-E6 is labelled with Venus and tracked (Assay 2). **Experiment ID 4 =** Counts of images for assays where 007-F8 is labelled with Venus and 007-F10 is labelled with mCherry.

(Related to experimental timepoints for Figures 4, 5 and 6 and Supplementary Figures 1, 2)

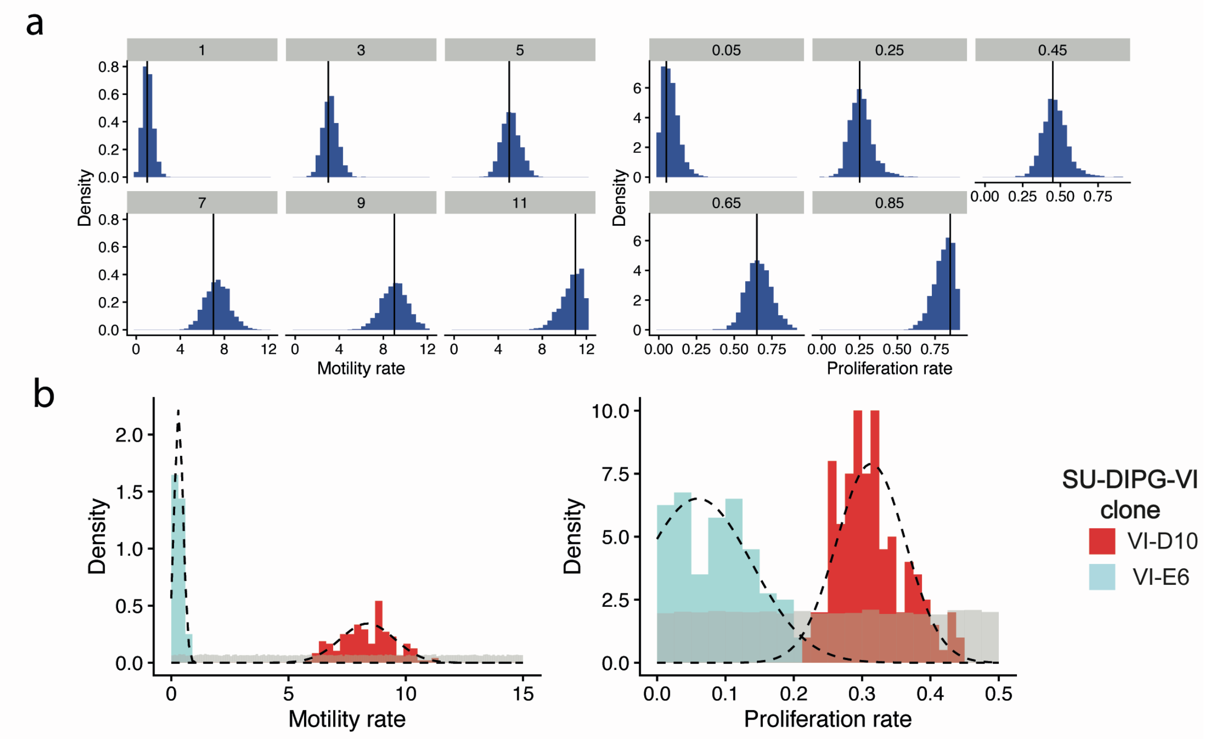

Supplementary figure 1: Recovery of invasion rates for SU-DIPG-VI clones VI-E6 and VI-D10 using area invaded with density of invaded area as summary statistics. a, Simulated recovery. **b,** Experimental recovery.

(Related to Figure 4)

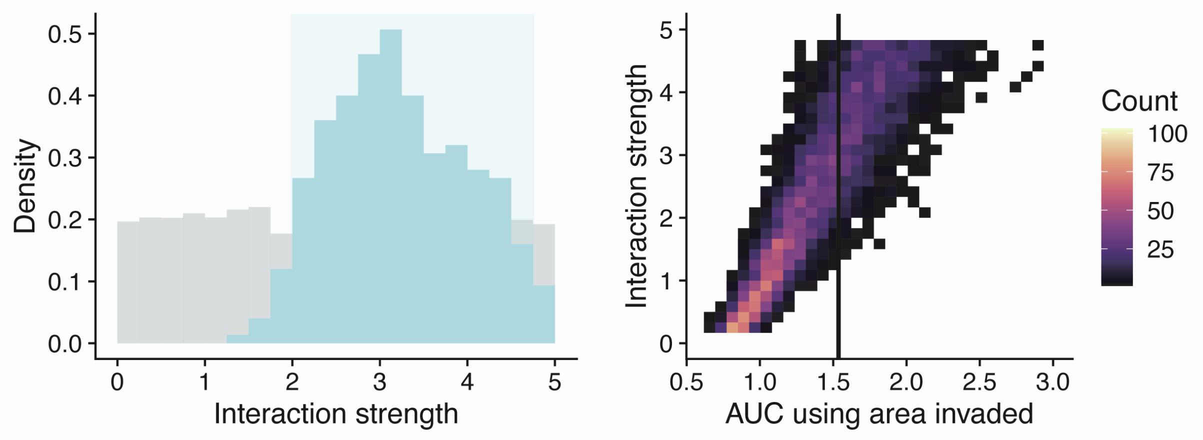

Supplementary figure 2: Additional replicate validates the experimental recovery of a positive interaction observed for SU-DIPG-VI clone VI-E6.

(Related to Figure 5)

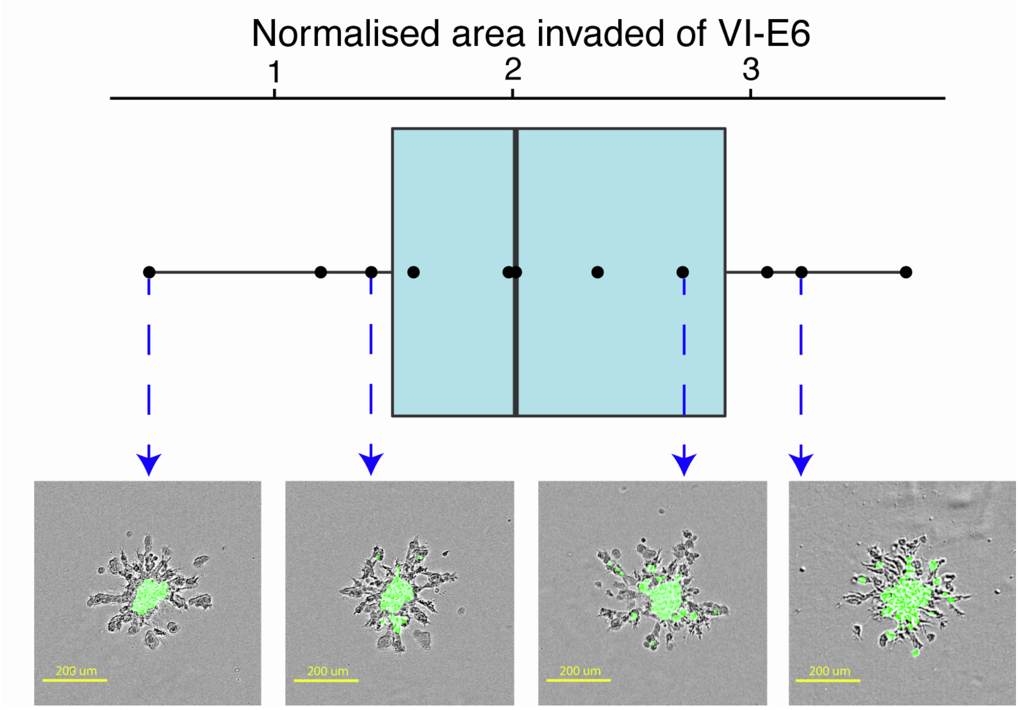

Supplementary figure 3: The interplay between spatial competition and positive interactions leads to variable outcomes in the invasive phenotype observed. Using VI-E6 (labelled in green) in a 50%-50% co-culture with VI-D10 as an illustrative example. Here the normalised area invaded of the green channel is measured**.** An overlay of the phase channel (showing all cells) with the green channel (showing only VI-E6 cells) demonstrates there is stochasticity in the interplay between interactions and spatial competition. In some replicates the spatial competition is able to prevent VI-E6 from invading, whilst in others VI-E6 appears free to invade in all directions (with minimal spatial restrictions). (Scale bars 200um).

(Related to Figure 5)

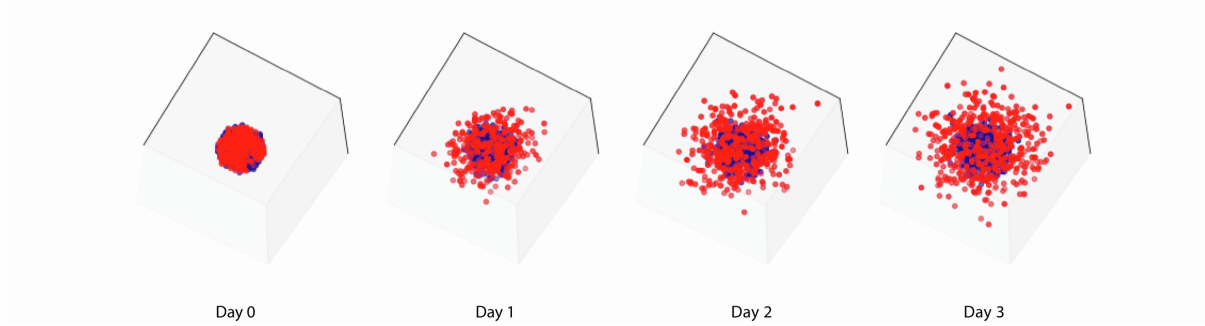

Supplementary figure 4: 3D visualisation of output from *in-silico* model of co-culture invasion. Two distinct cells are presented with identical proliferation rates (0.2 d/day) and differential motility rates. The population in red has a higher motility rate (10 x/day) than the blue population (1 x/day). **(Related to agent-based cellular automata in STAR Methods)**
