# Supplementary material for: Quantification of spatial subclonal interactions enhancing the invasive phenotype of pediatric glioma
Source: Cell Rep. 2022 Aug 30;40(9):111283. doi: 10.1016/j.celrep.2022.111283 (PMC9449134; doi:10.1016/j.celrep.2022.111283)
Supplement: Document S2. Article plus supplemental information [file mmc2.pdf]

Cell Reports

Article

Quantification of spatial subclonal interactions enhancing the invasive phenotype of pediatric glioma

Graphical abstract

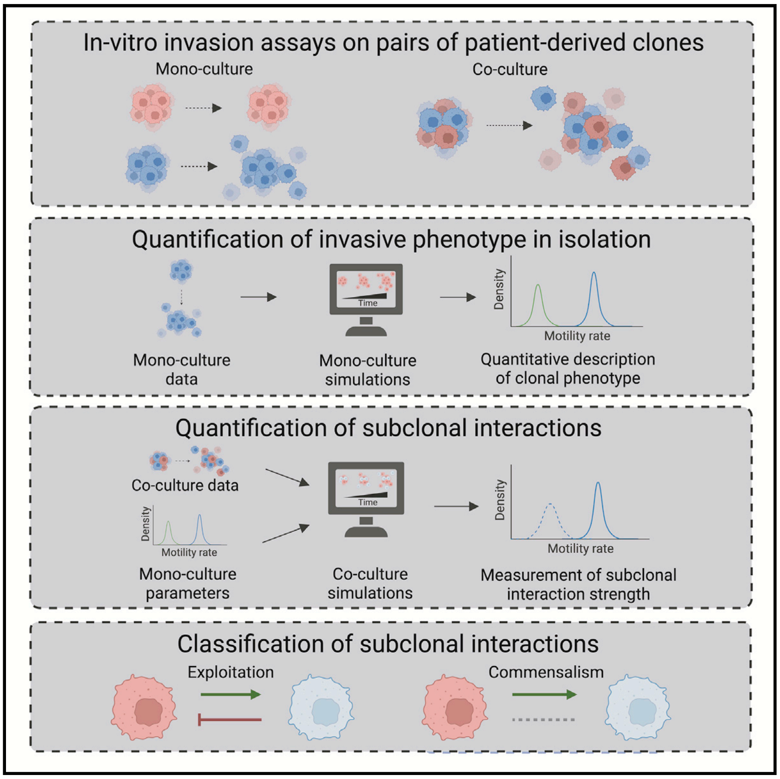

Authors

Haider Tari, Ketty Kessler, Nick Trahearn, Benjamin Werner, Maria Vinci, Chris Jones, Andrea Sottoriva

Correspondence

chris.jones@icr.ac.uk (C.J.), andrea.sottoriva@fht.org (A.S.)

In brief

Tari et al. explore interactions present between cancer cells and develop a methodology for their identification and quantification. Using a combination of computational modeling and experimental investigations, these interactions are demonstrated in pairs of single-cell-derived subclones isolated from independent patient tumors.

Highlights

- Explored the effects of interactions between cancer cells and their ability to spread
- Developed a computational and experimental framework to study spatial interactions
- Classified and quantified interactions present between two independent patient models
- Identified the presence of a commensalism interaction between DIPG subclones

Tari et al., 2022, Cell Reports *40*, 111283
August 30, 2022 © 2022 The Author(s).
https://doi.org/10.1016/j.celrep.2022.111283

CellPress
OPEN ACCESS

Article

Quantification of spatial subclonal interactions enhancing the invasive phenotype of pediatric glioma

Haider Tari,[1,2,6] Ketty Kessler,[2,6] Nick Trahearn,[1] Benjamin Werner,[3] Maria Vinci,[4] Chris Jones,[2,7,*] and Andrea Sottoriva[1,5,*]

[1]Evolutionary Genomics and Modelling Lab, Centre for Evolution and Cancer, The Institute of Cancer Research, London, UK
[2]Glioma Team, The Institute of Cancer Research, London, UK
[3]Barts Cancer Institute, Queen Mary University of London, London, UK
[4]Department of Haematology/Oncology, Cell and Gene Therapy, Bambino Gesù Children's Hospital-IRCCS, Rome, Italy
[5]Research Centre for Computational Biology, Human Technopole, Milan, Italy
[6]These authors contributed equally
[7]Lead contact
*Correspondence: chris.jones@icr.ac.uk (C.J.), andrea.sottoriva@fht.org (A.S.)
https://doi.org/10.1016/j.celrep.2022.111283

SUMMARY

Diffuse midline gliomas (DMGs) are highly aggressive, incurable childhood brain tumors. They present a clinical challenge due to many factors, including heterogeneity and diffuse infiltration, complicating disease management. Recent studies have described the existence of subclonal populations that may co-operate to drive pro-tumorigenic processes such as cellular invasion. However, a precise quantification of subclonal interactions is lacking, a problem that extends to other cancers. In this study, we combine spatial computational modeling of cellular interactions during invasion with co-evolution experiments of clonally disassembled patient-derived DMG cells. We design a Bayesian inference framework to quantify spatial subclonal interactions between molecular and phenotypically distinct lineages with different patterns of invasion. We show how this approach could discriminate genuine interactions, where one clone enhanced the invasive phenotype of another, from those apparently only due to the complex dynamics of spatially restricted growth. This study provides a framework for the quantification of subclonal interactions in DMG.

INTRODUCTION

Pediatric-type diffuse high-grade glioma, including diffuse midline glioma (DMG) such as diffuse intrinsic pontine gliomas (DIPGs), are a highly heterogeneous group of tumors with no effective treatments (Jones et al., 2017; Jones and Baker, 2014; Mackay et al., 2017). DMG, in particular, is characterized by a highly invasive phenotype that results in extensive infiltration of the brain parenchyma. This phenotype, coupled with the critical region within which these tumors originate, makes surgical resection difficult and leads to poor prognosis. In a recent study, Vinci et al. (2018) demonstrated the role of intra-tumoral heterogeneity (ITH) in the phenotypic severity of these tumors and implicated subclonal interactions as a potential driver of disease.

ITH is the natural consequence of an evolutionary process driven by random mutation, neutral drift, and non-random positive and negative selection (Turajlic et al., 2019). Moreover, as pediatric malignancies maintain a remnant of the differentiation program, cell signaling leading to interactions between lineages of cells or subclones has also been described (Azzarelli et al., 2018; Behjati et al., 2021; Jessa et al., 2019; Vinci et al., 2018). Evidence of subclonal interactions in other cancer types have been explored (Marusyk et al., 2014; Massagué and Obenauf, 2016; Tabassum and Polyak, 2015). However, these interactions remain difficult to quantify, and experimental observations are subject to bias and unaccounted confounding factors, such as spatial constraints and lack of mechanistic models applied to the data to test different alternative hypotheses.

Subclonal interactions can be studied through the lens of evolution and ecology, which seeks to understand the dynamics of a particular population within its environment and in relation to others. The most evident negative interaction between populations is competition for space and resources, leading to Darwinian selection (Christiansen and Loeschcke, 1990). There are also other forms of interactions such as amensalism, where the negative effect is only experienced by one population (Deines et al., 2017; Lidicker, 1979). Positive interactions instead lead to a population benefitting from the presence of another, and can arise in three varieties (Deines et al., 2017; Lidicker, 1979): mutualism, commensalism, and exploitation (Figure 1A). Mutualism is a two-sided benefit where two species to evolve to occupy complementary niches. Although it may be unlikely that two cancer subclones concomitantly evolve this form of adaptation by two independent subclones in the short timescales of a growing malignancy compared with millions of years in natural species, this has been suggested as a potential avenue for the survival of

This is an open access article under the CC BY license (http://creativecommons.org/licenses/by/4.0/).

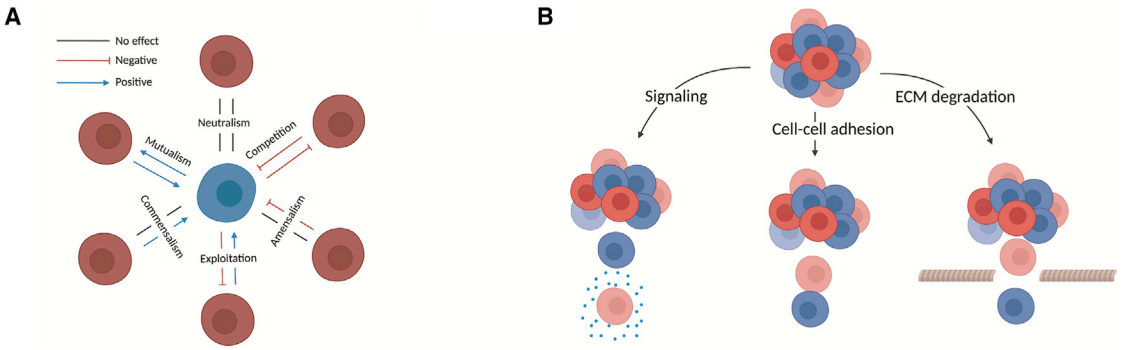

Figure 1. Schematic of the classification and nature of spatial subclonal interactions
(A) Illustration of interactions between sub-populations characterized by the effect one population has on another. The effect can be positive, negative, or neutral.
(B) Potential biological mechanisms through which spatial subclonal interactions can lead to enhanced invasion.

heterogeneous subclones (Axelrod et al., 2006). Commensalism, where a species benefits from the presence of another without affecting it, is instead potentially more likely as it only requires one population to provide an interaction that other populations can benefit from. An example is the production of extracellular signaling through secreted factors, which can lead to "public goods" dynamics, where all cells in the environment benefit from the subclone producing the signaling molecule (Archetti, 2016; Axelrod et al., 2006). Finally, exploitation interactions confer a positive benefit to one population at a cost to another, which, in the context of cancer, where turnover is considerably faster than in species, can lead to extinction. In multicellular organisms, this could be seen from the emergence of "cheaters" that violate the cooperative structure previously present (Aktipis and Maley, 2017). Indeed, examples of positive interactions have been observed in tumors, such as colon cancer, where paracrine amphiregulin production by treatment-resistant clones confers resistance to sensitive clones (Hobor et al., 2014). There have also been some studies that have found interactions driving tumor initiation (Polyak and Marusyk, 2014), metastasis (Marusyk et al., 2014), and cell growth (Cleary et al., 2014).

Mathematical modeling of cellular population dynamics allows simulating different interaction models to assess whether the different types of interaction fit the real data. There is a vast literature of mathematical models in cancer (Altrock et al., 2015; Gerlee and Nelander, 2012, 2016; Rockne et al., 2019; Stanková et al., 2019; Swanson et al., 2011; Zhang et al., 2017); however, these are not very often applied to data directly with the intent of inferring biology. Computational and mathematical modeling approaches are powerful, and, when used together with statistical inference, the resulting conclusions are measurable and translatable to further exploration (Zhang et al., 2017). Furthermore, although seminal studies have modeled interactions with evolutionary game theory in terms of growth advantage of subclones (Stanková et al., 2019), these approaches are largely non-spatial, and are not therefore suitable to capture the spatial patterns of invasion that we want to study in pediatric gliomas.

In DMG, we are interested in understanding whether there are cellular interactions driving its most problematic phenotype: the diffuse pattern of invasion. Subclonal interactions have been linked to an enhanced or diminished invasive capacity of an individual, with cooperation between subclones demonstrated in *Drosophila* between Ras^{V12} and $scrib^{-}$ clones (Wu et al., 2010). Much of the focus around subclonal interactions revolves around the growth rate of clones, with little attention devoted to the effects on collective cellular invasion. There are multiple biological mechanisms via which this interaction could occur: cell-cell adhesion leading to the co-invasion of cells, extracellular matrix degradation allowing for cells lacking this ability to escape, or paracrine signaling (Figure 1B) (Vinci et al., 2018). In this study, we focus on measuring interactions affecting collective cellular invasion.

This study focuses on a set of primary glioma cell lines derived from patients during rapid autopsies. The lines have been thoroughly characterized at the molecular and phenotypic levels. Importantly, from these cell lines, subclones with distinct molecular features and invasion characteristics have been isolated in a previous study by Vinci et al. (2018). In this study, clones were isolated from patient-derived cell lines with distinct genetics and phenotypes, with VI-D10 and VI-E6 isolated from SU-DIPG-VI and clones 007-F8 and 007-F10 isolated from HSJD-DIPG-007. Here we integrate co-culture *in vitro* invasion assays with a spatial computational modeling framework to quantify the presence, or lack of thereof, of spatial interactions affecting the invasive phenotype of a subclonal population. In this study, inference is divided in two sections; first, the phenotype of a pure population is quantified. This is achieved by coupling data from *in vitro* assays and *in silico* simulations to infer the distribution of parameters that quantitatively describe the phenotype of a population. The second part involves using *in silico* simulations to understand the effect of interactions of the invasion of a population. Here, the inference from mono-culture assays allows for the normalization of *in silico* simulations. The resulting computational model is leveraged to analyze co-culture assays to determine a quantitative description of the interactions present between two subclonal populations (Figure 2A).

RESULTS

Combining invasion assays with computational modeling to measure cellular interactions

The experimental design used in this study relies on growing tumor spheroids embedded in gelatinous matrix designed

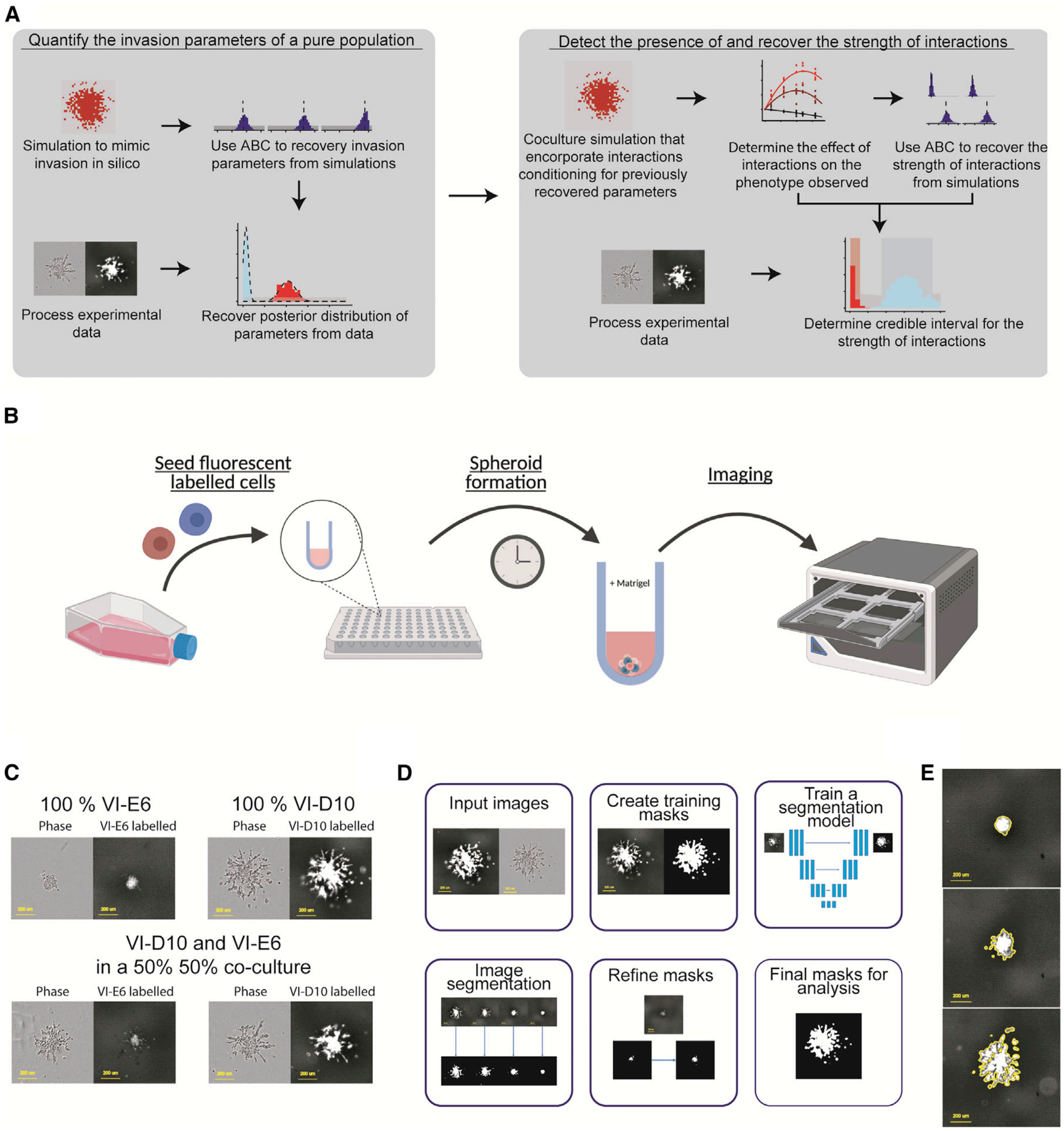

Figure 2. Combining computational modeling with invasion assays to measure cellular interactions

(A) We first quantify the invasion parameters of a pure population with computational inference previously validated *in silico*. We ensure we recovery the right parameters from simulated data, before applying the inference to real data from mono-cultures. We then detect and quantify the strength of interactions using the same approach, first with *in silico* inference and then inference on the real data, modeling different types and intensity of subclonal interactions. Inference on real data generates a credible interval of the true interaction strength.

(B) An illustration of the labeled co-culture experiments with spheroids embedded in an extracellular matrix and imaged over time.

(C) Images of pure and mixed cultures using phase and fluorescent channel to highlight the effectiveness of labeling in distinguishing sub-populations (scale bar, 200 μm).

(D) Deep learning image analysis framework for segmentation of cellular populations (scale bar, 200 μm).

(E) Images highlighting the result of image processing, with an outline of the binary mask (yellow) overlaid on the green fluorescence channel (scale bar, 200 μm).

to mimic the extracellular matrix and thus the conditions affecting tumor cells in patient tumors. We grow these distinct clones in mono-cultures and in co-cultures at various ratios to assess the differences in the invasive phenotype intrinsic to each clone, as well as the differences observed when cultured together (Figure 2A). Cells are seeded to form spheroids, which are then encapsulated in the extracellular matrix. These invading spheroids are imaged every 24 h using both phase and red/green fluorescence imaging (Figure 2B).

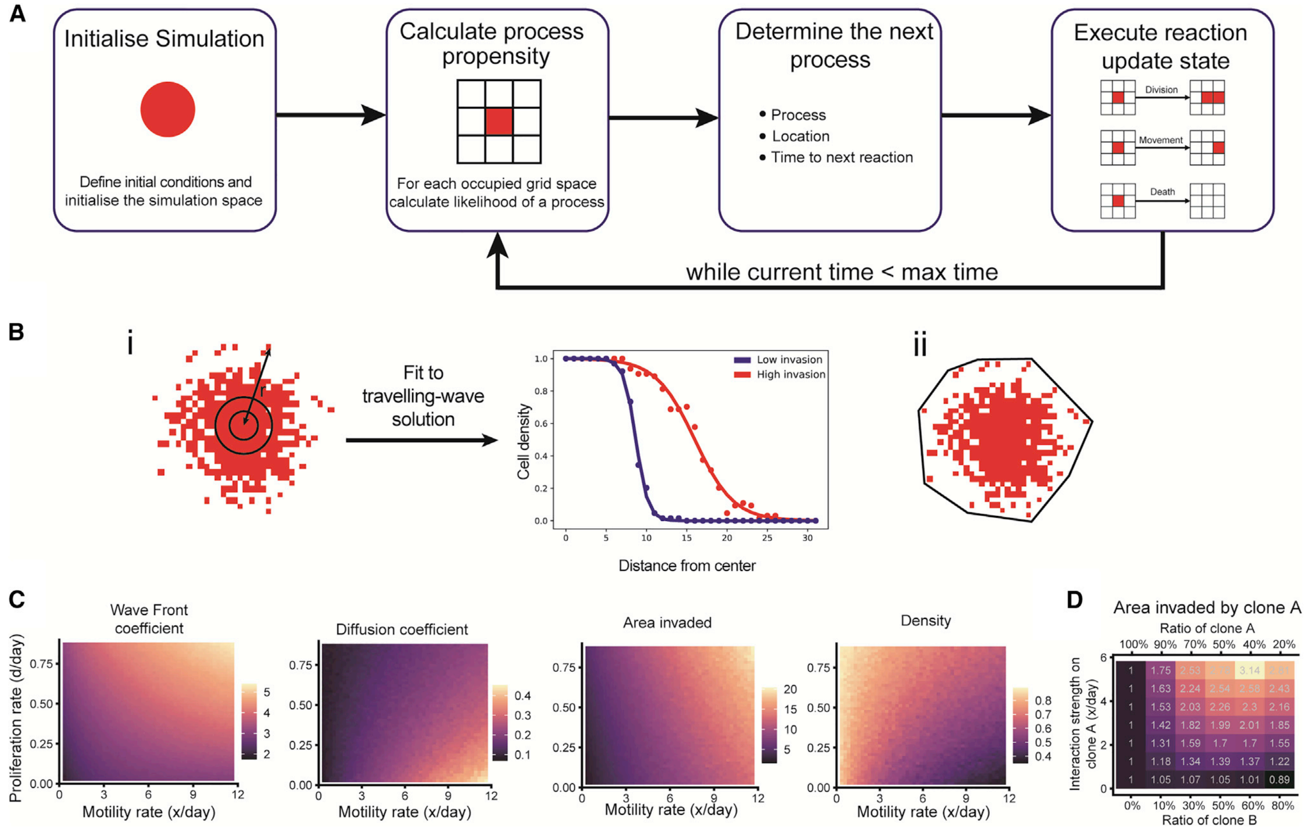

Figure 3. Simulating cellular invasion

(A) Illustration of simulation flow.

(B) Summary statistics used to measure invasion: (i) traveling-wave solution to reaction-diffusion equations fit to the spatial configuration of cells, (ii) convex hull area.

(C) Measurements of pure population invasion, detailing the effect of proliferation and motility rates on the phenotype observed.

(D) Highlighting the effect of introducing interactions on the phenotype observed. As the positive interaction strength increases, the area invaded relative to the pure culture also increases in a co-culture.

Fluorescent imaging is used to identify individual subclonal populations within a co-culture. This approach requires effective fluorescent marker expression and detection, and this is validated through the strong agreement of phase-contrast and fluorescent imaging channels for a mono-culture (Figure 2C). The 50:50 co-cultures of VI-E6 and VI-D10 demonstrated the effectiveness in the fluorescent channel providing subclonal resolution (Figure 2C). We used a tile-based deep learning image segmentation algorithm to process images from *in vitro* experiments. This algorithm created binary masks that represent the presence or absence of a subclone at a particular location within an image. Here a neural network is trained with a subset of images where the desired (ground truth) segmentation masks are provided (Figure 2D). These final masks are then compared with the original image to ensure accurate segmentation (Figure 2E). The final binary masks generated from this algorithm can be used to process spatial summary statistics, which are used for parameter inference using simulations.

We used a spatial simulator based on a 3D cellular automata (CA) model to simulate cellular invasion *in silico*. This model was used to generate spatial realizations that were compared with the data contained in experimental images. We used approximate Bayesian computation (ABC) to infer parameter values from experimental observations, by comparing binary masks generated from segmentation with simulation realizations, thus quantifying the biological characteristics of cell cultures.

We start by inferring parameters for a monoclonal population, to then create a null model of invasion in co-culture conditions. From this baseline model, we introduce subclonal interactions to test deviations from the null expectation. We determine a method of recovering the strength of interactions *in silico* and apply this method to *in vitro* data to detect the presence of and measure the strength of interactions in our experimental system (Figure 2A). The performance of parameter inference at each stage can be verified *in silico* to ensure consistent recovery of parameters from simulated data (see STAR Methods for details).

Simulating cellular invasion

We simulate cellular invasion using an agent-based model (Figure 3A). A similar approach has been used in previous studies to simulate glioma invasion (Gerlee and Nelander, 2012). The

CellPress
OPEN ACCESS

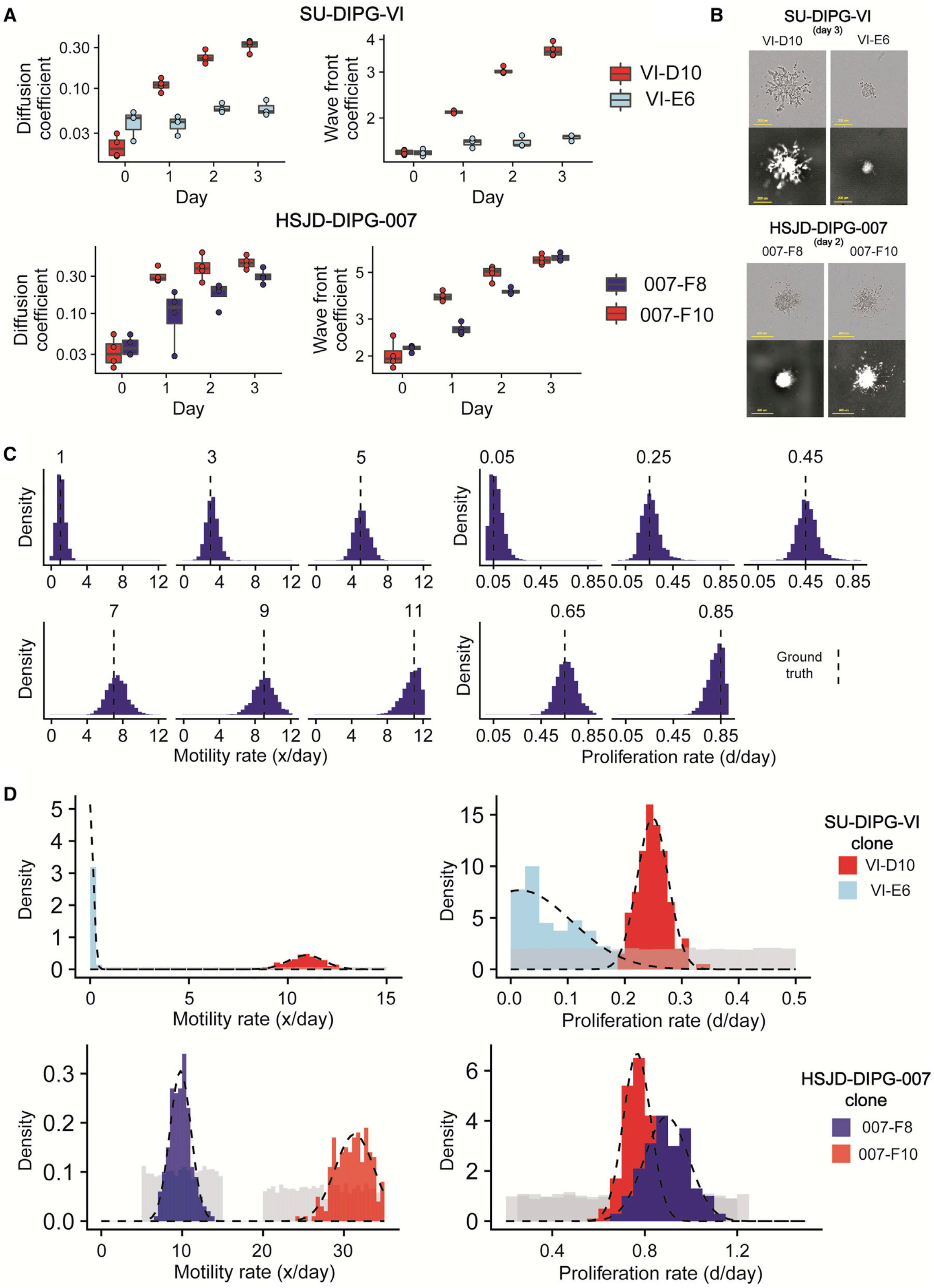

(legend on next page)

phenotype of each cell is described by two parameters: growth and movement. We use Gillespie's stochastic simulation algorithm (Gillespie, 1977) to generate simulations with temporal dynamics comparable with real data. (Figure 3A). Without an experimental metric to track cell death, we have assumed cell death is a fixed proportion (10%) of the proliferation rate to allow for cell turnover while ensuring positive growth. The model takes input parameters for the proliferation rate, describing rate of replication of a clone, and motility rate, describing the rate at which a clone moves around the environment. The unit of the proliferation rate is the number of divisions per day (d/day) and the unit of the motility rate is the number of cell widths, denoted as x, traveled (in any direction) per day (x/day).

We explored the resulting stochastic simulations to describe the invasion dynamics and generate summary statistics to quantify parameters. In order to make meaningful comparisons between experimental and simulated data, a set of summary statistics is required to reduce the dimensionality of the data. The first summary statistic proposed takes inspiration from previous studies of glioma invasion (Gerlee and Nelander, 2016), by using the traveling-wave solutions of a reaction-diffusion equation in order to find diffusion and wave-front coefficients, which describe how disperse a spatial configuration of cells is and how large the core of an invading spheroid is, respectively (Figure 3B, panel i). The coefficients we are using describe a static description of the configuration of cells, thus are not directly interpretable as reaction-diffusion equation coefficients. The diffusion coefficient (cell density/distance) describes how steep the slope of the wave is, whereas the wave-front coefficient (distance) describes distance of the initial high-density region of the wave. Other measures used are the area invaded (with units distance^2) and cellular density of invaded area (cell number/distance^2), we are able summarize the extent of invasion, while density seeks to provide information on the dispersion of the area invaded (Figure 3B, panel ii).

For a culture of a pure population with a fixed proliferation rate, the effect of increasing the motility rate is to increase the wave-front coefficient, diffusion coefficient, and area invaded while the density decreases (Figure 3C). This is not surprising as a population moving faster, while growing at the same rate, will occupy a larger area with the same number of total cells, leading to lower cellular density. On the other hand, keeping the motility rate fixed while increasing the proliferation rate causes wave-front coefficient, the area invaded, and the density to increase while the diffusion coefficient decreases (Figure 3C). Most importantly, the density and diffusion coefficient measurements have positive contours, while the wave-front coefficient and area invaded have negative contours, suggesting that a combination of these measurements can provide complementary information to recover both rates (Figure 3C).

Finally, looking at a model with interactions, we see the effect of an interaction on the invasive phenotype of co-culture seeding ratios. In co-culture conditions, as the interaction strength is increased, the value of the area invaded, normalized to the pure culture observation, increases, and therefore the invasive phenotype is enhanced (Figure 3D).

Measuring invasion in a mono-culture population

To assess the presence and strength of interactions present between distinct subclones, it is essential to understand the phenotype of each subclone in isolation. The two clones isolated from the tumor SU-DIPG-VI, VI-E6 and VI-D10, display a differential invasive phenotype. VI-D10 displays a stronger invasive phenotype, in comparison with VI-E6, as demonstrated by the diffusion and wave-front coefficients increasing faster (Figure 4A). This is evident in the images, which show a larger core and more dispersion of cells (Figure 4B). Similarly, between the two clones isolated from the tumor HSJD-DIPG-007, 007-F8 and 007-F10, 007-F10 displays a stronger invasive phenotype initially (Figures 4A and 4B). We do, however, observe a plateau in the invasion of 007-F10 after day 2.

We used ABC inference on *in silico* data to validate the accuracy of parameter recovery. This analysis is performed across a range of sample motility and proliferation rates to show there is consistent recovery of the ground truth (Figure 4C). We also tested the robustness of the inference using other summary statistics (Figure S1).

Applying this methodology to the binary masks generated from *in vitro* invasion assays, the posterior distributions for the proliferation and motility rates we recovered for SU-DIPG-VI clones VI-E6 and VI-D10 are consistent with the observation that VI-D10 displays significantly more invasion than VI-E6. The posterior distribution of proliferation and motility rates are then fitted to a distribution (truncated normal distributions), allowing for the quantification of the phenotype of each of our clones, and the use of these distributions as priors for our co-culture models (Figures 4D and S1). This allows for the propagation of uncertainty, which often is lost when inferring a single value and is crucial in avoiding skewing further inference.

We have also characterized the invasion parameters in a mono-culture for the clones 007-F8 and 007-F10 from the bulk tumor HSJD-DIPG-007, showing that 007-F8 displays a similar rate of proliferation but a substantially reduced motile phenotype compared with 007-F10 (Figure 4D).

Measuring cellular interactions between distinct clones in co-cultures

Using the posterior distributions recovered for mono-culture populations, we are able parameterize co-culture simulations. By assigning motility and proliferation rates that are drawn

Figure 4. Measuring the parameters that govern invasion of a monoclonal population

(A) Summary statistics calculated for experimental images from SU-DIPG-VI clones VI-E6 and VI-D10 and HSJD-DIPG-007 clones 007-F8 and 007-F10 demonstrates a clear difference in the invasive phenotype.

(B) Images show the differences in the invasion of spheroids (scale bars, 200 μm and 400 μm for SU-DIPG-VI and HSJD-DIPG-007 respectively).

(C) Posterior distribution of simulated recovery of sample data with ground truth highlighted by a dashed vertical line.

(D) Posterior distribution of parameters from experimental images, with truncated normal distributions fitted (dashed line). The prior distribution (gray) represents the distribution from the simulation parameters were drawn from (summary of number of experimental replicates in Table S2).

CellPress
OPEN ACCESS

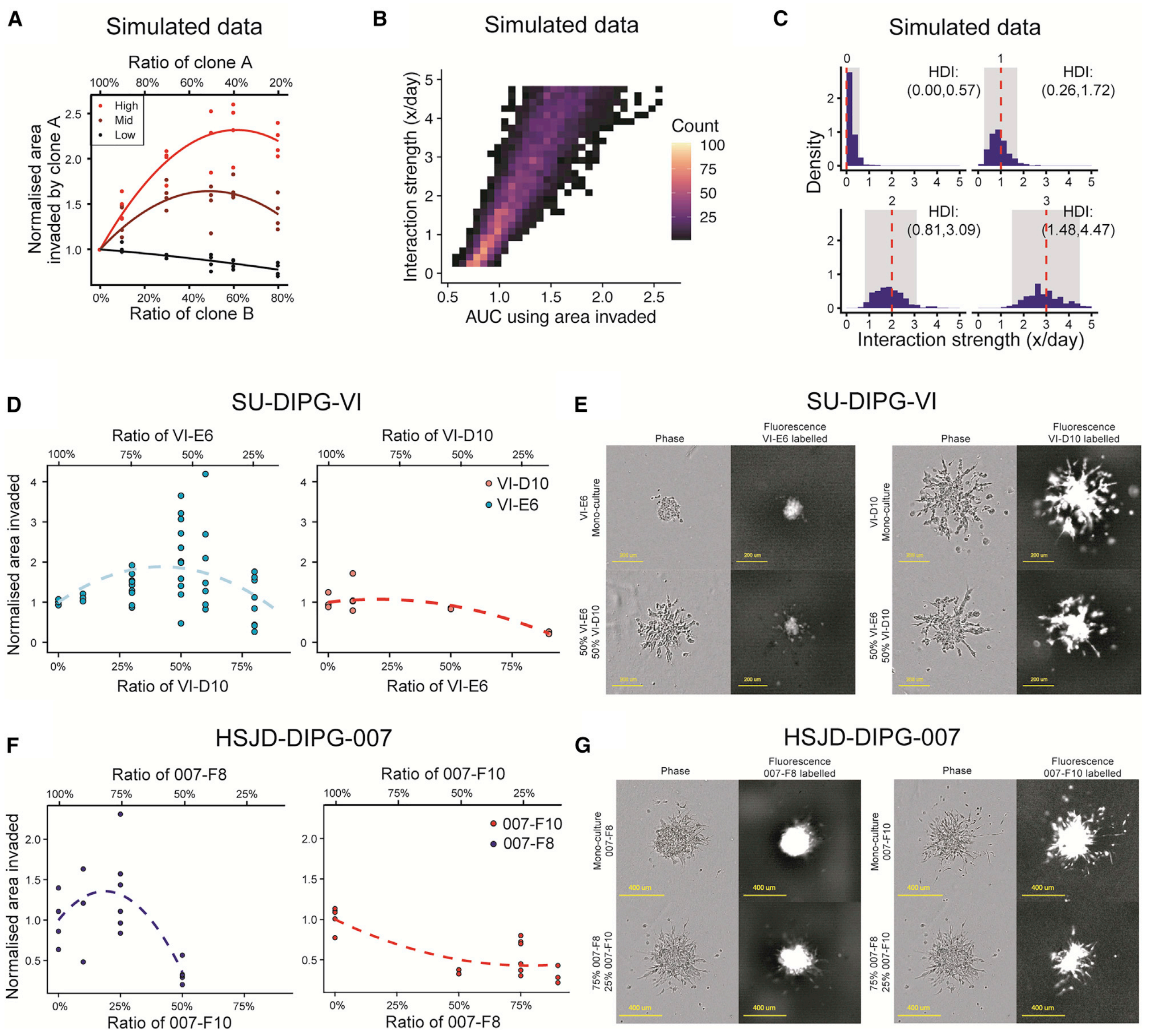

Figure 5. Summary statistics used to measure cellular interactions between distinct clones with differential invasion

(A) Summary statistic of invaded area of a clone normalized to its mono-culture observation for different interaction strengths.

(B) Linear relationship between area under curve and interaction strength.

(C) Posterior distribution of simulated data aimed at *in silico* recovery of the ground truth (red dashed line), with credible interval (gray).

(D) Summary statistics on experimental data from day 3 demonstrates the parabolic relationship for VI-E6 but not for VI-D10.

(E) Images demonstrate that VI-D10 displays lower invasion in a co-culture than in isolation, while VI-E6 displays enhanced invasion in a co-culture. Phase images show similar invasion between the two sets of co-culture images (scale bar, 200 μm).

(F) Summary statistics on experimental data from day 2 demonstrates the parabolic relationship for 007-F8 but not for 007-F10. In 007-F8 we see a similar parabolic relationship; however, there is a decrease to the level of 007-F10 in the 75:25 (007-F8:007-F10) ratio, while 007-F10 is strictly decreasing with a plateau.

(G) Images demonstrate that 007-F10 displays lower invasion in a co-culture than in isolation, while 007-F8 displays enhanced invasion in a co-culture. Phase images show that there is similar invasion in the two sets of co-culture images (scale bar = 400 μm) (summary of number of experimental replicates in Table S2).

from our posterior distributions, we can then focus on the effect that the interaction parameter has on the phenotype observed.

Based on simulations from a range of different interaction strengths, from low to high, we explored how an interaction affects the invasive phenotype *in silico*. For weak/absent interactions, we can see that the normalized area of a clone decreases as its seeding ratio decreases (Figure 5A). This is intuitive, as there are fewer cells to grow and move thus the invasion is less pronounced. However, introducing a positive interaction leads to a parabolic relationship, with a peak that increases as the interaction strength increases. This shows how introducing a small proportion of another clone that provides a positive

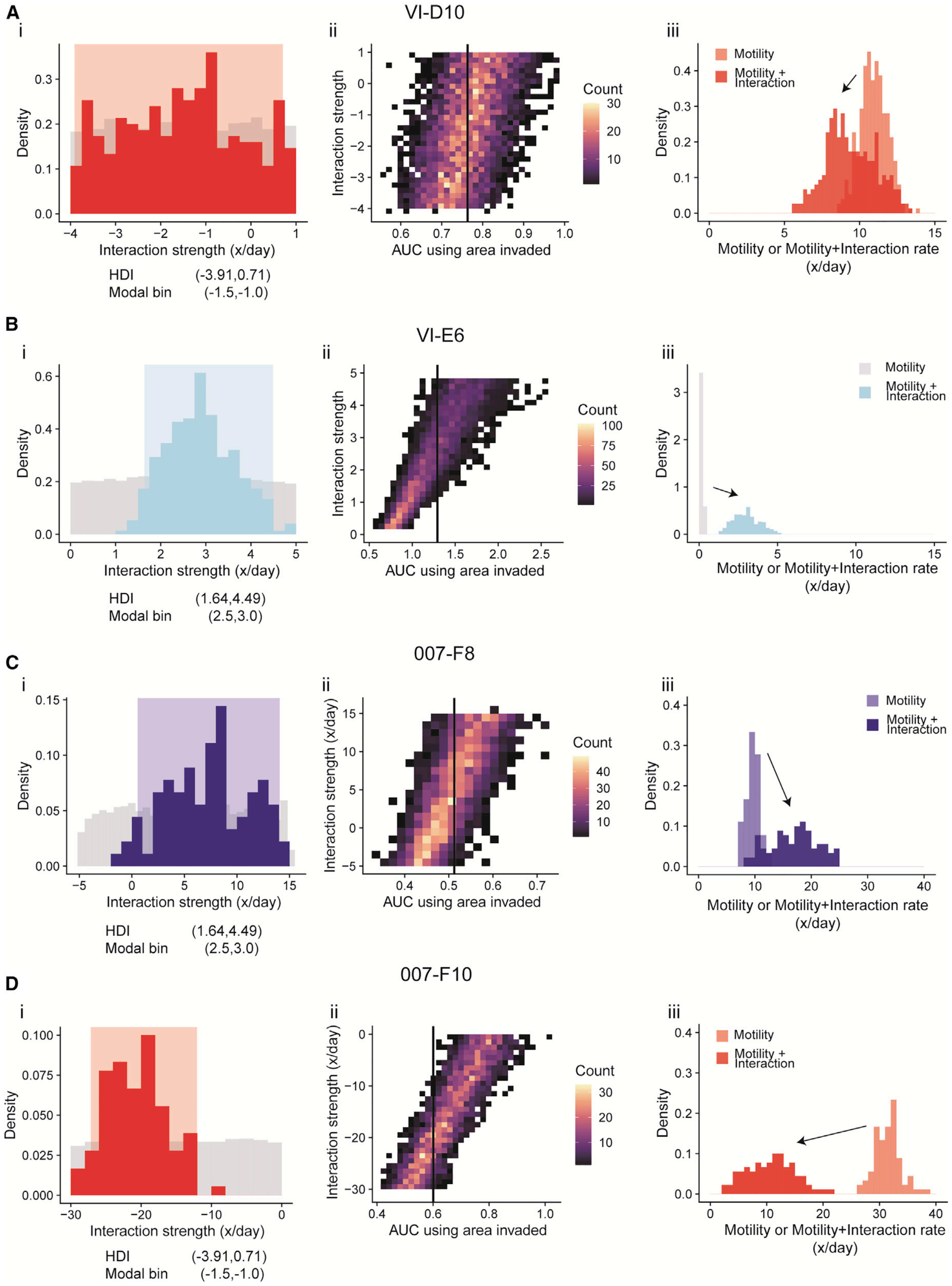

(legend on next page)

interaction leads to an increase the invasive phenotype. However, it is important to remember that the cells also experience competition with one another for both space and nutrients, such as growth factors in the cell culture medium, thus increasing the proportion of another clone by too much leads to a plateau in the area invaded followed by a decline as competition increases and counteracts the effect of interactions (Figure 5A).

Hence, increasing the interaction strength leads to the fitted curve becoming parabolic with an increasing peak. This can be summarized as increasing interaction strength corresponds with an increase in the area under the curve (AUC) (Figure 5B). This link suggests that AUC can be used as a summary statistic in ABC inference to recover the interaction strength. Indeed, applying this inference to quantify the interaction strength on simulated data, with AUC of the fitted curve as a summary statistic, leads to the correct recovery of the interaction strength parameter value. This relationship should allow for the recovery of the interaction strength by applying AUC as a summary statistic in ABC inference. We are also able to confirm the difference between zero and non-zero interactions, providing power to distinguish between their presence and absence. The ground truth is always contained in the credible interval for all samples, and thus there is confidence in the ability to recover the interaction strength using AUC as a summary statistic (Figure 5C).

In the experimental data, the VI-D10 population behaves similarly to that of no interactions, while E6 displays a parabolic phenotype, which suggests the presence of an interaction (Figure 5D). Indeed, experimental images demonstrate that VI-D10 invasion appears to be lower in the 50:50 (VI-E6:VI-D10) ratio compared with a mono-culture. However, on closer inspection of VI-E6, there are more cells that can escape and invade the surrounding matrix in the 50:50 (VI-E6:VI-D10) ratio compared with the mono-culture (Figure 5E). Similarly, we can see that, between the clones 007-F8 and 007-F10, 007-F8 displays a weak parabolic relationship that quickly declines to below 1 (the mono-culture normalized value) at a ratio seeded of 50:50 (007-F8:007-F10), while 007-F10 displays a significantly negative trend as the ratio seeded decreases (Figure 5F). Once again, closer inspection of images shows that indeed 007-F8 and 007-F10 have invaded a smaller distance at the 75:25 (007-F8:007-F10) ratio (Figure 5G).

By applying our computational inference framework to those experimental images, we then generated credible intervals for the biological parameters. For VI-D10, the credible interval overlaps with 0, with a modal interaction strength interval of (−1.5, −1.0) (Figure 6A). This indicates that there is insufficient evidence to suggest there is any interaction received by VI-D10. More interestingly, in the case of VI-E6, the credible interval for all replicates does not contain 0, with one replicate demonstrating a modal interaction strength interval of (2.5, 3.0) (Figures 6B and S2). The evidence indicates that a model with interactions better explains the data than a model without, coupled with the reproducibility of the interaction strength across multiple experiments strongly indicated that VI-E6 receives a measurable commensal interaction from VI-D10.

Further investigating the clones 007-F8 and 007-F10, we see that 007-F8 displays a positive interaction strength with a credible interval that does not overlap with 0 and a modal interaction strength interval of (8, 9) (Figure 6C), while F10 displays a credibly negative interaction strength with a modal interaction strength of (−20, −18) (Figure 6D). The evidence indicates that a model with interactions better explains the data than a model without, with the posterior distributions suggesting that 007-F8 displays an exploitative interaction on 007-F10.

We can also infer the maximal possible effect interactions can have on the motility of a clone by comparing the distribution of the motility rate plus interaction strength against just the motility rate alone. We observe that the VI-E6 demonstrates a noticeable positive shift in the distribution, while VI-D10 has largely overlapping distributions with a slight negative shift (Figures 6A and 6B). The same conclusions can be drawn for 007-F8 and 007-F10, with 007-F10 showing a noticeable negative shift in the distribution, while 007-F8 has slightly overlapping distributions with a noticeable positive shift (Figures 6C and 6D).

We see larger stochasticity in our experimental data compared with simulations, and this is particularly noticeable for measurements of VI-E6. This can be explained by the interplay between interactions and competition, where there is the potential for spatial competition to mask the effect of any interaction. This occurs when VI-D10 is able to quickly engulf VI-E6, thus limiting VI-E6 in terms of finding space to invade. We demonstrated this by overlaying the green fluorescence channel on top of the phase images, to highlight the possible scenarios, from complete freedom to invade in all directions to complete spatial restriction from being engulfed (Figure S3).

Here we have demonstrated, from two pairs of subclonal populations derived from two different bulk tumors, the presence of a commensal interaction as well as an exploitative interaction.

DISCUSSION

In this study, we present a quantitative methodology enabling the detection and measurement of positive spatial interactions that affect the invasive phenotype of subclonal populations. We applied this to a set of single-cell-derived clones from two DMG autopsy patients. These findings help understanding the role of ITH, a phenomenon linked to the adverse prognosis for

Figure 6. Quantification of interaction strengths across two pairs of subclones from two patient-derived models

These pairs of subclones are VI-E6 with VI-D10 and 007-F8 with 007-F10.

(A) VI-D10 receives a neutral interaction (when cultured with VI-E6).

(B) VI-E6 receives a positive interaction (when cultured with VI-D10).

(C) 007-F8 receives a positive interaction (when cultured with 007-F10).

(D) 007-F10 receives a negative interaction (when cultured with 007-F8). (i) Posterior distributions of the interaction strength received by a clone, (ii) simulated data plotted with the experimental AUC as a vertical line, (iii) distributions highlighting the maximal effects of interactions (summary of number of experimental replicates in Table S2).

patients, particularly in the case of pediatric-type high-grade gliomas (Vinci et al., 2018). The ability to detect and measure subclonal interactions can open avenues for testing and validating treatments that seek to contain tumors by disrupting positive interactions and promoting negative interactions.

We demonstrated a measurable change in the invasive phenotype of a subset of clones used in this study, which was observable only when grown in the presence of other distinct clones. This is particularly of note in the context of disease development and progression as it reveals the implications of tumoral heterogeneity and subclonal interactions on tumors. DMG is characterized by a highly invasive phenotype that results in infiltration of critical regions of the brain parenchyma, so understanding the factors that result in the phenotype is crucial in further understanding the disease. We measured the effect of heterogeneity on the cellular invasion through the modeling of co-cultures of single-cell-derived clones with results showing both positive and negative interactions. Most notably, clone VI-E6 displayed negligible invasive potential in a mono-culture; however, this was significantly enhanced when in a co-culture with VI-D10, which is another clone from the same tumor. The effect on the motility of VI-D10 was minimal and, as a result, we demonstrated the presence of a spatial interaction that can be classified as commensalism. This demonstrated that the phenotype of individual clone in a tumor cannot be considered in isolation and the effect of subclonal interactions is a potential avenue for enhancing disease aggression. We also demonstrated the presence of an exploitative interaction where clone 007-F8 benefited from a higher motility rate in a co-culture, while clone 007-F10 suffers with a fall in its motility rate. These results demonstrated the role of subclonal interactions in altering the characteristics of tumor cells and add a layer of complexity to our understanding of how tumors grow and develop.

The most obvious extension to our methodology would be to include the inference of cell death rates. We have set the death rate to 10% of the proliferation rate to ensure turnover and growth without extinction; however, this choice could be adjusted if the rate could be directly measured. Through the use cell death markers, such as using Annexin V and flow cytometry to quantify the proportion of dead cells, this rate could be measured and further enhance the predictive value of stochastic simulations. Another factor to explore is the emergence of quiescence where a clone enters a reversible state of non-proliferation; this is highlighted by the "go or grow" hypothesis in gliomas. Here cells can either move through the microenvironment or divide through phenotypic switching (Hatzikirou et al., 2012). Modeling this switching in phenotype can provide more detailed insight into the invasive dynamics (Godlewski et al., 2010). However, based on the short durations of the assays and low cell numbers used, it is unlikely that cell death was a key driver of the phenotypic changes observed in this study. Further expansion to include the effect of therapeutics that would induce cell death would inevitably require an adjustment to the experimental design.

In the case of this study, a key addition would be to introduce mechanistic modeling to determine the nature of interactions and find the causal factor. The clones used in this study were established and characterized in our previous study, where we highlighted the phenotypic consequences in co-cultures (Vinci et al., 2018). This characterization revealed a small number of important somatic mutations that genotypically defined the subclones, as well as differences in the expression of some key genes associated with chemokine signaling. These latter observations represent an attractive candidate mechanism for the interactions we have observed, whereby treatment of non-motile clones with the pro-migratory chemokines secreted by the motile cells enhanced these phenotypes *in vitro*.

There are a multitude of different avenues for interactions to exhibit themselves, such as being pulled via cell-cell adhesion (Janiszewska et al., 2020; Reher et al., 2017) or modulation of chemokine signaling (Helbig et al., 2003; Manu et al., 2011; Marusyk et al., 2014; Tabassum and Polyak, 2015). This will require the creation of assays tailored to detecting these interactions, which should be matched with computational models that aim to do the same. Examples of such designs could be achieved through the use of conditioned medium for long-range interactions (Chang et al., 2012; Liu et al., 2019a), or several homogeneous spheroids from a number of different clones can be seeded in the same well and the invasive phenotype of each spheroid can be correlated to the distance from one another. Experimental designs such as these could help determine the distance across which interactions occur. Exploring models to recreate the interplay between spatial competition and interactions will help broaden the understanding of how likely a particular clone is to be engulfed and how this may change with the seeding ratio and strength of interaction.

Spatial interactions are largely unexplored in cancer, and little has been done in particular for those affecting cellular invasion, with much of the literature focusing on the proliferative interactions. This study presents an approach to tackle spatial interactions by demonstrating the ability to detect positive and negative interactions as well as estimate their strengths across biological replicates; however, there is significant room for further developments. Detecting interactions is merely the initial step; the ultimate goal should be to use our understanding of such interactions to improve patient outcomes through enhanced prediction of the trajectory of disease and therapeutic interventions. A crucial avenue of exploration is the role of the tumor microenvironment on cellular invasion (Altorki et al., 2019; Marusyk et al., 2014; Tabassum and Polyak, 2015). Numerous studies have highlighted the role of microenvironmentally induced tumor spread and metastasis, such as those demonstrating the role of cancer-associated fibroblasts (Gaggioli et al., 2007; Liu et al., 2019b) or macrophages (Chen et al., 2019; Lin et al., 2019). Future studies designed to expand on the findings of this work could explore the role of the tumor microenvironment on cellular invasion as well as an understanding of the translation of discoveries from *in vitro* studies to *in vivo* and clinical settings. This could be achieved through the use of *in vivo* models to simulate the tissue structure faced by tumors, organotypic brain slice cultures to introduce microenvironmental features (Chadwick et al., 2015), and adjusted *in vitro* models to introduce microenvironmental factors. The microenvironment, however, is a highly complex system, thus initial exploration is necessary to identify the factors that are the most pertinent to the invasion phenotype observed. Finally, an application of measuring the response of spatial

interactions to therapeutic interventions can be performed using drug screens that seek to derive a relationship between dose and interaction strength.

This study combined computational modeling with the study of intra-tumoral interactions between subclones and created a model trained from two pairs of distinct patient-derived cell lines that are well characterized phenotypically and genotypically. This model can therefore be applied to more cell lines and subclones, and potentially more cancer types. However, the experimental techniques would require the ability to distinguish between a larger number of distinct populations in a co-culture. Using different combinations of fluorescent tags simultaneously could be used to create a system that could track many uniquely labeled populations (Pericoli et al., 2020). For example, with a choice of three tags (such as red, green, and blue fluorescent proteins; Weber et al., 2008) applying combinatorics, up to seven distinguishable populations can be created. More interestingly, mass cytometry technology presents the opportunity to tag over 50 populations simultaneously when combined with protein barcoding (Wroblewska et al., 2018). Jin et al. (2020) demonstrated the ability to tag many human cell lines and track their metastatic potential in a co-culture *in vivo* environment using similar technology. From this study, inspiration can be drawn and a more complex ecosystem of interacting cancer cells could be modeled; however, such an expansion will create a heightened computational burden. In this study, we highlighted the presence of spatial subclonal interactions, and it is clear that future explorations integrating a greater degree of complexity should be considered.

Advances in technologies such as *in situ* spatial profiling of tissues or imaging mass cytometry present an interesting avenue for further exploration of data with a finer resolution, and this will allow for the extension of such approaches to a more diverse experimental system, with increased subclonal heterogeneity and inclusions of factors from the tumor microenvironment. Integrating enhanced microenvironmental complexity can be achieved through the use of brain slices, such as those demonstrated by Venkatesh et al. (2015). These experimental advances are crucial in extending the inference methodology applied in this study to system more closely replicating tumor dynamics in patients.

The model used in this study provides a crucial stepping stone in detecting spatial subclonal interactions that have seldom been explored. Detecting and quantifying the presence of such interactions indicated that greater attention should be drawn to such phenomena. Our approach allows for the identification of interactions, which can in turn be an indication of where resources and time should be spent to further explore the causes of such interactions.

Limitations of the study

Some limitations of this current study must be acknowledged. As discussed previously, this study focuses on the subclonal interactions between two pairs of single-cell-derived cell lines. The approach of using pairs of subclones was deliberate as we sought to establish the presence of spatial subclonal interactions, whose presence becomes convoluted as complexity increases. However, understanding more complex dynamics of interactions is crucial and must be investigated further by building on the approaches demonstrated in this study. Additionally, features of the tumor microenvironment were largely absent from this model, besides the inclusion of subclonal heterogeneity and the use of an extracellular matrix. Through the profiling of the relevant tumor microenvironment, studies can seek to introduce layers of complexity, such as adding the presence of stromal cells into a co-culture or extending experimental systems into an *in vivo* setting. Finally, the optimization of labeling techniques is essential in distinguishing between clones, especially once model complexity is increased, and techniques such as imaging mass cytometry should be investigated.

STAR★METHODS

Detailed methods are provided in the online version of this paper and include the following:

- KEY RESOURCES TABLE
- RESOURCE AVAILABILITY
  - Lead contact
  - Materials availability
  - Data and code availability
- EXPERIMENTAL MODEL AND SUBJECT DETAILS
  - Primary cell culture
- METHOD DETAILS
  - Study and experimental design
  - Cell culture
  - Cell doubling time
  - Cell labeling
  - Invasion assays
  - Cell imaging
  - Image segmentation
  - Genetic algorithm
  - Traveling-wave solution to a reaction-diffusion equation
  - Gillespie's stochastic simulation algorithm (SSA)
  - Agent based cellular automaton
- QUANTIFICATION AND STATISTICAL ANALYSIS
  - Bayesian computation
  - Validation of summary statistics

SUPPLEMENTAL INFORMATION

Supplemental information can be found online at https://doi.org/10.1016/j.celrep.2022.111283.

ACKNOWLEDGMENTS

This work was supported by Cancer Research UK (A23536). K.K. is supported by CHILDREN with CANCER UK. A.S. is supported by the Wellcome Trust (202778/B/16/Z) and Cancer Research UK (A22909). We acknowledge funding from the National Institute of Health (NCI U54 CA217376) to A.S. This work was also supported by a Wellcome Trust award to the Centre for Evolution and Cancer (105104/Z/14/Z). M.V. is a Children with Cancer UK Fellow. Some illustrations were created using BioRender.com.

AUTHOR CONTRIBUTIONS

H.T., A.S., and C.J. conceived the study. H.T. carried out computational analysis. K.K. carried out experiments. M.V. provided regents. H.T., K.K., C.J., and A.S. wrote the manuscript. All authors read and approved the manuscript.

DECLARATION OF INTERESTS

The authors declare no competing interests.

INCLUSION AND DIVERSITY

One or more of the authors of this paper self-identifies as an underrepresented ethnic minority in science. One or more of the authors of this paper self-identifies as a member of the LGBTQ+ community.

Received: September 6, 2021
Revised: March 21, 2022
Accepted: August 8, 2022
Published: August 30, 2022

REFERENCES

Aktipis, A., and Maley, C.C. (2017). Cooperation and cheating as innovation: insights from cellular societies. Philos. Trans. R. Soc. Lond. B Biol. Sci. *372*, 20160421. https://doi.org/10.1098/rstb.2016.0421.

Altorki, N.K., Markowitz, G.J., Gao, D., Port, J.L., Saxena, A., Stiles, B., McGraw, T., and Mittal, V. (2019). The lung microenvironment: an important regulator of tumour growth and metastasis. Nat. Rev. Cancer *19*, 9–31. https://doi.org/10.1038/s41568-018-0081-9.

Altrock, P.M., Liu, L.L., and Michor, F. (2015). The mathematics of cancer: integrating quantitative models. Nat. Rev. Cancer *15*, 730–745. https://doi.org/10.1038/nrc4029.

Archetti, M. (2016). Cooperation among cancer cells as public goods games on Voronoi networks. J. Theor. Biol. *396*, 191–203. https://doi.org/10.1016/j.jtbi.2016.02.027.

Axelrod, R., Axelrod, D.E., and Pienta, K.J. (2006). Evolution of cooperation among tumor cells. Proc. Natl. Acad. Sci. USA *103*, 13474–13479. https://doi.org/10.1073/pnas.0606053103.

Azzarelli, R., Simons, B.D., and Philpott, A. (2018). The developmental origin of brain tumours: a cellular and molecular framework. Development *145*, dev162693. https://doi.org/10.1242/dev.162693.

Behjati, S., Gilbertson, R.J., and Pfister, S.M. (2021). Maturation block in childhood cancer. Cancer Discov. *11*, 542–544. https://doi.org/10.1158/2159-8290.CD-20-0926.

Chadwick, E.J., Yang, D.P., Filbin, M.G., Mazzola, E., Sun, Y., Behar, O., Pazyra-Murphy, M.F., Goumnerova, L., Ligon, K.L., Stiles, C.D., and Segal, R.A. (2015). A brain tumor/organotypic slice Co-culture system for studying tumor microenvironment and targeted drug therapies. J. Vis. Exp., e53304. https://doi.org/10.3791/53304.

Chang, Y.-H., Lee, S.-H., Liao, I.-C., Huang, S.-H., Cheng, H.-C., and Liao, P.-C. (2012). Secretomic analysis identifies Alpha-1 antitrypsin (A1AT) as a required protein in cancer cell migration, invasion, and pericellular fibronectin assembly for facilitating lung colonization of lung adenocarcinoma cells. Mol. Cell. Proteomics *11*, 1320–1339. https://doi.org/10.1074/mcp.M112.017384.

Chen, Y., Song, Y., Du, W., Gong, L., Chang, H., and Zou, Z. (2019). Tumor-associated macrophages: an accomplice in solid tumor progression. J. Biomed. Sci. *26*, 78. https://doi.org/10.1186/s12929-019-0568-z.

Chkhaidze, K., Heide, T., Werner, B., Williams, M.J., Huang, W., Caravagna, G., Graham, T.A., and Sottoriva, A. (2019). Spatially constrained tumour growth affects the patterns of clonal selection and neutral drift in cancer genomic data. PLoS Comput. Biol. *15*, e1007243. https://doi.org/10.1371/journal.pcbi.1007243.

Christiansen, F.B., and Loeschcke, V. (1990). Evolution and competition. In Population Biology: Ecological and Evolutionary Viewpoints, K. Wöhrmann and S.K. Jain, eds. (Springer Berlin Heidelberg), pp. 367–394. https://doi.org/10.1007/978-3-642-74474-7_13.

Cleary, A.S., Leonard, T.L., Gestl, S.A., and Gunther, E.J. (2014). Tumour cell heterogeneity maintained by cooperating subclones in Wnt-driven mammary cancers. Nature *508*, 113–117. https://doi.org/10.1038/nature13187.

Deines, P., Lachnit, T., and Bosch, T.C.G. (2017). Competing forces maintain the Hydra metaorganism. Immunol. Rev. *279*, 123–136. https://doi.org/10.1111/imr.12564.

Gaggioli, C., Hooper, S., Hidalgo-Carcedo, C., Grosse, R., Marshall, J.F., Harrington, K., and Sahai, E. (2007). Fibroblast-led collective invasion of carcinoma cells with differing roles for RhoGTPases in leading and following cells. Nat. Cell Biol. *9*, 1392–1400. https://doi.org/10.1038/ncb1658.

Gerlee, P., and Nelander, S. (2012). The impact of phenotypic switching on glioblastoma growth and invasion. PLoS Comput. Biol. *8*, e1002556. https://doi.org/10.1371/journal.pcbi.1002556.

Gerlee, P., and Nelander, S. (2016). Travelling wave analysis of a mathematical model of glioblastoma growth. Math. Biosci. *276*, 75–81. https://doi.org/10.1016/j.mbs.2016.03.004.

Gillespie, D.T. (1977). Exact stochastic simulation of coupled chemical reactions. J. Phys. Chem. *81*, 2340–2361. https://doi.org/10.1021/j100540a008.

Godlewski, J., Bronisz, A., Nowicki, M.O., Chiocca, E.A., and Lawler, S. (2010). microRNA-451: a conditional switch controlling glioma cell proliferation and migration. Cell Cycle *9*, 2742–2748.

Hatzikirou, H., Basanta, D., Simon, M., Schaller, K., and Deutsch, A. (2012). 'Go or Grow': the key to the emergence of invasion in tumour progression? Math. Med. Biol. *29*, 49–65. https://doi.org/10.1093/imammb/dqq011.

He, K., Zhang, X., Ren, S., and Sun, J. (2015). Deep residual learning for image recognition. Preprint at arXiv. https://doi.org/10.48550/arXiv.1512.03385.

Helbig, G., Christopherson, K.W., Bhat-Nakshatri, P., Kumar, S., Kishimoto, H., Miller, K.D., Broxmeyer, H.E., and Nakshatri, H. (2003). NF-K B promotes breast cancer cell migration and metastasis by inducing the expression of the chemokine receptor CXCR4. J. Biol. Chem. *278*, 21631–21638. https://doi.org/10.1074/jbc.M300609200.

Hobor, S., Van Emburgh, B.O., Crowley, E., Misale, S., Di Nicolantonio, F., and Bardelli, A. (2014). TGFα and amphiregulin paracrine network promotes resistance to EGFR blockade in colorectal cancer cells. Clin. Cancer Res. *20*, 6429–6438. https://doi.org/10.1158/1078-0432.CCR-14-0774.

Janiszewska, M., Primi, M.C., and Izard, T. (2020). Cell adhesion in cancer: beyond the migration of single cells. J. Biol. Chem. *295*, 2495–2505. https://doi.org/10.1074/jbc.REV119.007759.

Jessa, S., Blanchet-Cohen, A., Krug, B., Vladoiu, M., Coutelier, M., Faury, D., Poreau, B., De Jay, N., Hébert, S., Monlong, J., et al. (2019). Stalled developmental programs at the root of pediatric brain tumors. Nat. Genet. *51*, 1702–1713. https://doi.org/10.1038/s41588-019-0531-7.

Jin, X., Demere, Z., Nair, K., Ali, A., Ferraro, G.B., Natoli, T., Deik, A., Petronio, L., Tang, A.A., Zhu, C., et al. (2020). A metastasis map of human cancer cell lines. Nature *588*, 331–336. https://doi.org/10.1038/s41586-020-2969-2.

Jones, C., and Baker, S.J. (2014). Unique genetic and epigenetic mechanisms driving paediatric diffuse high-grade glioma. Nat. Rev. Cancer *14*, 651–661. https://doi.org/10.1038/nrc3811.

Jones, C., Karajannis, M.A., Jones, D.T.W., Kieran, M.W., Monje, M., Baker, S.J., Becher, O.J., Cho, Y.-J., Gupta, N., Hawkins, C., et al. (2017). Pediatric high-grade glioma: biologically and clinically in need of new thinking. Neuro Oncol. *19*, 153–161. https://doi.org/10.1093/neuonc/now101.

Lidicker, W.Z. (1979). A clarification of interactions in ecological systems. Bioscience *29*, 475–477. https://doi.org/10.2307/1307540.

Lin, Y., Xu, J., and Lan, H. (2019). Tumor-associated macrophages in tumor metastasis: biological roles and clinical therapeutic applications. J. Hematol. Oncol. *12*, 76. https://doi.org/10.1186/s13045-019-0760-3.

Liu, P., Kong, L., Jin, H., Wu, Y., Tan, X., and Song, B. (2019a). Differential secretome of pancreatic cancer cells in serum-containing conditioned medium reveals CCT8 as a new biomarker of pancreatic cancer invasion and metastasis. Cancer Cell Int. *19*, 262. https://doi.org/10.1186/s12935-019-0980-1.

Liu, T., Han, C., Wang, S., Fang, P., Ma, Z., Xu, L., and Yin, R. (2019b). Cancer-associated fibroblasts: an emerging target of anti-cancer immunotherapy. J. Hematol. Oncol. *12*, 86. https://doi.org/10.1186/s13045-019-0770-1.

Mackay, A., Burford, A., Carvalho, D., Izquierdo, E., Fazal-Salom, J., Taylor, K.R., Bjerke, L., Clarke, M., Vinci, M., Nandhabalan, M., et al. (2017). Integrated

CellPress
OPEN ACCESS

molecular meta-analysis of 1, 000 pediatric high-grade and diffuse intrinsic pontine glioma. Cancer Cell *32*, 520–537.e5. https://doi.org/10.1016/j.ccell.2017.08.017.

Manu, K.A., Shanmugam, M.K., Rajendran, P., Li, F., Ramachandran, L., Hay, H.S., Kannaiyan, R., Swamy, S.N., Vali, S., Kapoor, S., et al. (2011). Plumbagin inhibits invasion and migration of breast and gastric cancer cells by downregulating the expression of chemokine receptor CXCR4. Mol. Cancer *10*, 107. https://doi.org/10.1186/1476-4598-10-107.

Marusyk, A., Tabassum, D.P., Altrock, P.M., Almendro, V., Michor, F., and Polyak, K. (2014). Non-cell-autonomous driving of tumour growth supports subclonal heterogeneity. Nature *514*, 54–58. https://doi.org/10.1038/nature13556.

Massagué, J., and Obenauf, A.C. (2016). Metastatic colonization by circulating tumour cells. Nature *529*, 298–306. https://doi.org/10.1038/nature17038.

Pericoli, G., Petrini, S., Giorda, E., Ferretti, R., Ajmone-Cat, M.A., Court, W., Conti, L.A., De Simone, R., Bencivenga, P., Palma, A., et al. (2020). Integration of multiple platforms for the analysis of multifluorescent marking technology applied to pediatric GBM and DIPG. Int. J. Mol. Sci. *21*, 6763. https://doi.org/10.3390/ijms21186763.

Polyak, K., and Marusyk, A. (2014). Cancer: clonal cooperation. Nature *508*, 52–53. https://doi.org/10.1038/508052a.

Reher, D., Klink, B., Deutsch, A., and Voss-Böhme, A. (2017). Cell adhesion heterogeneity reinforces tumour cell dissemination: novel insights from a mathematical model. Biol. Direct *12*, 18. https://doi.org/10.1186/s13062-017-0188-z.

Rockne, R.C., Hawkins-Daarud, A., Swanson, K.R., Sluka, J.P., Glazier, J.A., Macklin, P., Hormuth, D.A., Jarrett, A.M., Lima, E.A.B.F., Tinsley Oden, J., et al. (2019). The 2019 mathematical oncology roadmap. Phys. Biol. *16*, 041005. https://doi.org/10.1088/1478-3975/ab1a09.

Ronneberger, O., Fischer, P., and Brox, T. (2015). U-net: convolutional networks for biomedical image segmentation. In Medical Image Computing and Computer-Assisted Intervention – MICCAI 2015, Lecture Notes in Computer Science, N. Navab, J. Hornegger, W.M. Wells, and A.F. Frangi, eds. (Springer International Publishing), pp. 234–241. https://doi.org/10.1007/978-3-319-24574-4_28.

Stanková, K., Brown, J.S., Dalton, W.S., and Gatenby, R.A. (2019). Optimizing cancer treatment using game theory: a review. JAMA Oncol. *5*, 96–103. https://doi.org/10.1001/jamaoncol.2018.3395.

Swanson, K.R., Rockne, R.C., Claridge, J., Chaplain, M.A., Alvord, E.C., and Anderson, A.R.A. (2011). Quantifying the role of angiogenesis in malignant progression of gliomas: in silico modeling integrates imaging and histology. Cancer Res. *71*, 7366–7375. https://doi.org/10.1158/0008-5472.CAN-11-1399.

Tabassum, D.P., and Polyak, K. (2015). Tumorigenesis: it takes a village. Nat. Rev. Cancer *15*, 473–483. https://doi.org/10.1038/nrc3971.

Turajlic, S., Sottoriva, A., Graham, T., and Swanton, C. (2019). Resolving genetic heterogeneity in cancer. Nat. Rev. Genet. *20*, 404–416. https://doi.org/10.1038/s41576-019-0114-6.

Venkatesh, H.S., Johung, T.B., Caretti, V., Noll, A., Tang, Y., Nagaraja, S., Gibson, E.M., Mount, C.W., Polepalli, J., Mitra, S.S., et al. (2015). Neuronal activity promotes glioma growth through neuroligin-3 secretion. Cell *161*, 803–816. https://doi.org/10.1016/j.cell.2015.04.012.

Vinci, M., Gowan, S., Boxall, F., Patterson, L., Zimmermann, M., Court, W., Lomas, C., Mendiola, M., Hardisson, D., and Eccles, S.A. (2012). Advances in establishment and analysis of three-dimensional tumor spheroid-based functional assays for target validation and drug evaluation. BMC Biol. *10*, 29. https://doi.org/10.1186/1741-7007-10-29.

Vinci, M., Box, C., and Eccles, S.A. (2015). Three-dimensional (3D) tumor spheroid invasion assay. J. Vis. Exp., e52686. https://doi.org/10.3791/52686.

Vinci, M., Burford, A., Molinari, V., Kessler, K., Popov, S., Clarke, M., Taylor, K.R., Pemberton, H.N., Lord, C.J., Gutteridge, A., et al. (2018). Functional diversity and cooperativity between subclonal populations of pediatric glioblastoma and diffuse intrinsic pontine glioma cells. Nat. Med. *24*, 1204–1215. https://doi.org/10.1038/s41591-018-0086-7.

Weber, K., Bartsch, U., Stocking, C., and Fehse, B. (2008). A multicolor panel of novel lentiviral "gene ontology" (LeGO) vectors for functional gene analysis. Mol. Ther. *16*, 698–706. https://doi.org/10.1038/mt.2008.6.

Wroblewska, A., Dhainaut, M., Ben-Zvi, B., Rose, S.A., Park, E.S., Amir, E.-A.D., Bektesevic, A., Baccarini, A., Merad, M., Rahman, A.H., and Brown, B.D. (2018). Protein barcodes enable high-dimensional single-cell CRISPR screens. Cell *175*, 1141–1155.e16. https://doi.org/10.1016/j.cell.2018.09.022.

Wu, M., Pastor-Pareja, J.C., and Xu, T. (2010). Interaction between RasV12 and scribbled clones induces tumour growth and invasion. Nature *463*, 545–548. https://doi.org/10.1038/nature08702.

Zhang, J., Cunningham, J.J., Brown, J.S., and Gatenby, R.A. (2017). Integrating evolutionary dynamics into treatment of metastatic castrate-resistant prostate cancer. Nat. Commun. *8*, 1816. https://doi.org/10.1038/s41467-017-01968-5.

STAR★METHODS

KEY RESOURCES TABLE

REAGENT or RESOURCE	SOURCE	IDENTIFIER
Bacterial and virus strains		
Subcloning DH5α™ Competent cells	Invitrogen™	#18265017
One Shot™ Stbl3™ Chemically Competent E. coli	Invitrogen™	#C737303
Lentivirus Lego iC2	This paper	N/A
Lentivirus Lego V2	This paper	N/A
Biological samples		
HSJD-DIPG-007 (male, 9.9 years old)	SU: Michelle Monje, Stanford University	N/A
SU-DIPG-VI (female, 7 years old)	HSJD: Angel Montero Cascaboso, Hospital San Joan de Deu, Barcelona	N/A
Chemicals, peptides, and recombinant proteins		
Trans-Lentiviral shRNA Packaging system	Dharmacon	#TLP5912
Lipofectamine2000	Invitrogen	#11668030
LentiX Concentrator	Takara	#631231
NucLight Red BacMam 3.0	Sartorius	#4621
NucLight Green BacMam 3.0	Sartorius	#4622
Primocin	Invivogen	#ant-pm
Plasmocin	Invivogen	#ant-mpt
Dulbecco's Modified Eagles Medium: Nutrient Mixture F12	Thermo Fisher	#11330-032
Neurobasal-A Medium	Thermo Fisher	#10888-022
HEPES Buffer Solution	Thermo Fisher	#15630-080
MEM sodium pyruvate solution	Thermo Fisher	#11360-070
MEM nonessential amino acids solution	Thermo Fisher	#11140-050
Glutamax-I Supplement	Thermo Fisher	#35050-061
B-27 Supplement Minus Vitamin A	Thermo Fisher	#12587-010
Human-EGF	Shenandoah Biotech	#100-26
Human-FGF-basic	Shenandoah Biotech	#100-146
Human-PDGF-AA	Shenandoah Biotech	#100-16
Human-PDGF-BB	Shenandoah Biotech	#100-18
Heparin Solution, 0.2%	StemCell Technologies	#07980
Laminin	Bio-Techne	#3446-005-01
Accutase	Sigma	# A6964
Matrigel Basement Membrane Matrix, LDEV-free	Corning	#354234
Critical commercial assays		
Plasmid maxi kit	Qiagen	#12362
Cell Titer-Glo® Luminescent Cell Viability	Promega	#G7571 and #G7572
Deposited data		
Deposited data - Experimental	This paper	https://doi.org/10.17632/mszhzrm4dd.1
Deposited data - Simulation	This paper	https://doi.org/10.17632/mszhzrm4dd.1
Deposited data - Images	This paper	https://doi.org/10.17632/mszhzrm4dd.1
Deposited code – Computational simulator	This paper	https://doi.org/10.5281/zenodo.6835901
Deposited code - Image analysis	This paper	https://doi.org/10.5281/zenodo.6835901

(*Continued on next page*)

Continued

REAGENT or RESOURCE	SOURCE	IDENTIFIER
Experimental models: Cell lines		
HEK293T	Gibco™	#A35347
Recombinant DNA		
Lego-iC2	Addgene	#27345
Lego-V2	Addgene	#27340
Software and algorithms		
CUDA C	Nvidia	CUDA Toolkit 11.4.0
MATLAB	https://www.mathworks.com/products/matlab.html	R2021b
R	https://www.r-project.org/	3.5.3
Python3	https://www.python.org	3.7
Incucyte S3 live-cell analysis system	Sartorius	N/A
OpenCV	Opencv.org	4.4.0.44
PyTorch	Pytorch.org	1.10

RESOURCE AVAILABILITY

Lead contact

Further information and requests for resources and reagents should be directed to and will be fulfilled by the lead contact, Chris Jones (chris.jones@icr.ac.uk).

Materials availability

Materials generated in this study are available upon request from the lead contact, subject to an MTA.

Data and code availability

- Statement about data: Data is available via Mendeley data at https://doi.org/10.17632/mszhzrm4dd.1.
- Statement about code: Code for computational analysis is available at https://doi.org/10.5281/zenodo.6835901.
- Any additional information required to reanalyze the data reported in this paper is available from the lead contact upon request.

EXPERIMENTAL MODEL AND SUBJECT DETAILS

Primary cell culture

In this study, two primary patient-derived cell lines: SU-DIPG-VI (female, 7 years old) (*H3F3A* ^{K27M}, *TP53* $^{p.R175H\ \&\ p.E198*}$, *MYC* ^{amp}) and HSJD-DIPG-007 (male, 9.9 years old) (*H3F3A* ^{K27M}, *ACVR1* ^{R206H}, *PPM1D*$^{p.P428fs\blacktriangle}$, *PIK3CA*$^{p.H1047R}$). Use of human material covered by Multiregional Research Ethics Committee approval 18/LO/0514, all samples collected under full informed consent.

METHOD DETAILS

Study and experimental design

In *in-vitro* cell culture there is considerable variability between the phenotype of a cell across replicates, even when ensuring conditions are kept as consistent as possible. This represents a significant barrier in separating the signal present between interacting subclonal populations and noise from the variability present. To alleviate this issue, we conducted all mono-culture and co-culture assays of the same cell line in parallel. This allows for much of the experimental noise to be eliminated as cells seeded will be from the same passage, at the same time and under near-identical cell culture conditions.

Cell culture

Patient-derived cultures SU-DIPG-VI and HSJD-DIPG-007 were grown in stem cell media consisting of Dulbecco's Modified Eagles Medium: Nutrient Mixture F12 (DMEM/F12), Neurobasal-A Medium, HEPES Buffer Solution 1M, sodium pyruvate solution 100nM, nonessential amino acids solution 10mM, Glutamax-I Supplement and penicillin Streptomycin solution (all Thermo Fisher, Loughborough, UK). The media was supplemented with B-27 Supplement Minus Vitamin A, (Thermo Fisher), 20ng/ml Human-EGF, 20ng/ml Human-FGF-basic-154, 20ng/ml Human-PDGF-AA, 20ng/ml Human-PDGF-BB (all Shenandoah Biotech, Warwick, PA, USA) and 2μg/ml Heparin Solution (0.2%, Stem Cell Technologies, Cambridge, UK) to constitute the complete media. Cells were incubated at 37°C, 5% CO2, 95% humidity and were refed at least twice weekly with complete media. Cell authenticity was verified using short

tandem repeat (STR) DNA fingerprinting. SU-DIPG-VI cells were maintained on laminin-coated flasks/plates at 10μg/ml (Bio-Techne, 3446-005-01), whereas HSJD-DIPG-007 cells were maintained as neurospheres. When the cells reached confluency, 90% surface area or 200μm neurosphere, cells were split into new flasks and/or plates depending on the assay required. Each split, cells were dissociated by enzymatic reaction using Accutase (Sigma, A6964) for 2-5min at 37°C then diluted into PBS before centrifugation at 1000rpm for 10min for neurospheres or 13000rpm for 5min for 2D cells. The cell pellet was resuspended with complete media to obtain a single suspension that can be assessed for cell count and viability using the automated cell counter Countess II FL (Invitrogen, AMQAX1000). Further details on human glioma lines used can be found on www.crukchildrensbraintumourcentre.org/research/resources/cell-line-repository/.

Cell doubling time

Cell doubling time was assessed by seeding 6000 and 4000 cells into 96-well back plates (Grainer, 655976) for SU-DIPG-VI and HSJD-DIPG-007 respectively. The cell viability was measured at different timepoints using Cell Titer-Glo (Promega, G7571 and G7572) following the manufacturer's instructions. We generated a growth curve from which we could calculate the doubling time using the readings from the two timepoints flanking the exponential phase. The doubling time results are presented in Table S1.

Cell labeling

Different clones were derived from the bulk of SU-DIPG-VI (VI-D10 and VI-E6) and HSJD-DIPG-007 (007-F8, 007-F10) as previously described in our previous publication (Vinci et al., 2018).

Each clone was stably labeled with the lentiviral "gene ontology" (LeGO) vectors (Weber et al., 2008). Transduction were performed using lentivirus encapsulated with the following plasmids: the plasmid Lego-iC2, mCherry expressing vector (#27345; Addgene) or the plasmid Lego-V2, Venus expressing vector (#27340; Addgene), allowing the fluorescent gene of interest to be integrated into the genome of the cells and expressed constitutively.

Briefly, each LeGo plasmid was transfected into HEK293T cells together with the Trans-Lentiviral shRNA Packaging System (#TLP5912; Dharmacon) helped by Lipofectamine (2000) (#11668030; Invitrogen). Forty-eight hours post-transfection viral particles in the supernatant were collected, filtered through Millex-HV 0.45um filter (#SLHVM33RS, Millipore), concentrated with LentiX Concentrator according to the manufacturer's instructions (#631231, Takara) then stored aliquoted at −80C. For a clonal selection, the transfected cells were single cell flow sorted into the inner 60 wells of 96 well plates ultra-low attachment round bottom (#7007, Corning) using a Beckman Coulter MoFlo in Class IIA2 biohazard containment hood. Cells were dropped in 100μL/well of complete media supplemented with 2X growth factors, Primocin (#ant-pm, InvivoGen), Plasmocin (#ant-mpt, InvivoGen), penicillin and streptomycin (Life Technologies). In order to enhance the fluorescent signal, clones were also labeled transiently with NucLight Red or Green BacMam 3.0 Reagent (#4621 and 4622 respectively; Sartorius (discontinued) before seeding for the assays.

A parallel two-colour system (red/green) was used for 007-F8 and 007-F10. For clones VI-E6 and VI-D10, the expression of the mCherry vector was not sufficient to be detected, thus a single color system (green) was used to track a single subclone: VI-E6 was expressing Venus in green and VI-D10 was expressing mCherry in red in some of the assays, and in some others VI-D10 was expressing Venus and VI-E6 was expressing mCherry. When the cells are seeded as co-culture, the cells were labelled first using the methods described above and then seeded with the different ratio as a mixed population. To fully evaluate their invasion ability without being biased by the labeling effect, we considered only the results from the green channel as shown in the Results section.

Invasion assays

We generated single spheres per well using an ultra-low attachment 96-well plate (Corning, 7007). Cells were seeded in 200μL of complete media at D0 as the following: 200 cells for SU-DIPG-VI bulk and 250 cells for VI-E6 and VI-D10; 150 cells for HSJD-DIPG-007 bulk and 150 cells for 007-F8 and 007-F10. They were centrifuged at 1300rpm for 5min and incubated at 37C, 5% CO_2, 95%. At D3, the neurospheres from the mono- and co-cultures, 6 replicates per condition, reached about 50-100μm diameter.

Invasion assays were performed as previously described (AU - Vinci et al., 2015; Vinci et al., 2018, 2012), with some modifications. A total of 100μL medium was removed from each well containing the single neurosphere. Cold Matrigel (Corning, 354234) was gently added at 100 μL/well and plates were incubated at 37°C, 5% CO_2, 95% humidity for 1hr. Once the Matrigel solidified, 100μL/well of culture medium was added on top and cells were incubated in normal condition in the IncucyteS3 for imaging throughout the length of the assay.

Cell imaging

Images are taken using the Incucyte S3 live-cell analysis system using the spheroid scanning module with a 4× objective. Images were taken of the phase, red and green imaging channels at intervals of 24h.

Image segmentation

First images were exported from the IncuCyte S3 with out of focus images discarded. To enhance the signal in the fluorescent channels a pre-processing step of contrast-limited adaptive histogram equalisation (CLAHE) was applied using a 5x5 pixel window, resulting in enhancement of the signal in the fluorescent channels.

We then prepared training data, to train a neural network to identify features from the images to segment on. Ground truth training masks of positive cells were produced by manual annotation. These masks were created for phase and any applicable fluorescence channels. A total of 120 masks were used for training distributed across four assays conducted in this study.

The ground truth masks were used to train a UNet (Ronneberger et al., 2015)-style segmentation, using ResNet-18(He et al., 2015) blocks pre-trained on ImageNet data for encoding. Training data was divided using an 80–20 training-validation split (with 96 training images and 24 validation images) and running the training process until the validation error converged. This trained model was used for the segmentation in the next step.

We then applied our segmentation model on the phase and fluorescent images, resulting in probability maps that indicate the probability that each pixel contains a cell. These probability maps are converted to binary masks by thresholding at a cutoff of 0.5, pixel with probability above this are determined to contain a cell.

The position of the microscope camera may change throughout the experiment. Thus, our time series image data must be registered into a common reference frame before cell movement can be compared across images. In order to do this, we used phase correlation to perform image registration, using the imregcorr function in MATLAB's Image Processing toolbox. This is performed using the phase channel to generate a transform which shifts an image in the x-y plane to fit the previously seen image. For each well we fixed the earliest image as the reference frame and applied this transform.

We then reviewed each binary mask to evaluate their accuracy. To reduce the potential of false positive pixel we required that a pixel would only be classed as a cell if it was positive on both a phase and fluorescence channel. This has the cost of increasing the proportion of pixels that are false negatives (meaning they are incorrectly labeled as not containing a cell when in fact they do. To address this, segmentation masks were overlaid on the original images to identify any under-segmentation. We compared masks with the original images and corrected inaccurate masks are corrected. The result of this process is a final binary mask used for further analysis.

Genetic algorithm

Summary statistics are combined using a weighted Euclidean distance between observed and simulated values. The optimal weighting of each summary statistic to this distance was determined using a genetic algorithm. The optimal weights minimised the squared distance between the ground truth and value of parameters recovered in the posterior distribution. This was carried out over a range of values with optimal recovery demonstrated in Figure 4C. Multiple simulations were initialised across a range of starting weights with the resulting fitness compared to ensure likely convergence to a global optimum. Analysis was produced using the GA package in R.

Traveling-wave solution to a reaction-diffusion equation

$$C(r) = \frac{1}{1 + e^{\frac{r-m}{2a}}}$$

where r is the distance from the centre of a spheroid, $C(r)$ is the cellular density at a distance r from the center, m is the wave front coefficient representing the growth of the dense core of a spheroid and a is the diffusion coefficient which represents the motility of cells.

Gillespie's stochastic simulation algorithm (SSA)

SSA was used to calculate the likelihood of each process and the time taken to the next process to occur in an agent-based cellular automaton. This was processed according to the study by Gillespie (1977) (Gillespie, 1977).

Agent based cellular automaton

A cellular automaton (CA) is a class of discrete models in computing, consisting of a finite-dimensional grid which each point in the grid representing a finite state and pre-determined rules that are followed. These models have been used extensively to study natural processing including models in oncology (Chkhaidze et al., 2019). Agent-based CA introduce distinct independent 'agents' that behave according to their own pre-defined set of rules.

We created a CA model on a three-dimensional grid with each point representing an empty space or containing a single cell. Each cell can undergo a pre-determined set of possibilities; divide into a neighbor grid point, move to a neighboring grid point (cells cannot move to an occupied grid point) or undergo cell death – in all cases using a (using a 3D Moore neighbourhood of length 1) (Figures 2A and S4). A cell can be assigned a distance to which it can push all neighbouring cells, this is called the proliferation aggression parameter (Chkhaidze et al., 2019). The aggression parameter is used when proliferation is chosen to occur and there is no space in the local neighbourhood. In this case if there is an empty space within distance defined by the aggression parameter, the cell will divide into the local neighborhood and push all other cells outwards to occupy the empty space.

Each of these processes occur at different rates and as the model is extended, we will have a distinct set of rates for each individual subpopulation we are modelling. To determine the next process to occur at any given time and the location at which this occurs we employed Gillespie's stochastic simulations algorithm (SSA) (Gillespie, 1977). This is a common choice and has been shown to

generate a statically consistent trajectory of stochastic equations, with a caveat of being computationally intensive. In this study the cell death rate was set to be proportional to the division rate (10% of division rate) as this will allow for turnover of cells but also reduce the computational requirements of the model when used for inference.

We initiated a simulation with cells set to a pre-determined radius around a central point (this radius was set to 5 pixels). From this state, we calculated the propensity for each process to occur. Next, we chose a random process and cell for the next process to occur, weighted for propensity (the likelihood of each process), as well as the time to next process using the total propensity. At this point we updated the state of the simulation by updating the current time and executing the process selected. This loop is repeated until the end time of the simulation is achieved; at set intervals we take a snapshot of the state of the simulation to use for analysis (Figure 2A). In all instances, when a snapshot of the simulation is saved, we create a 2D collapse of the 3D configuration (see STAR Methods) to be representative of the nature microscope images, which are 2D representations of a 3D system.

We modify our previous model to account for co-culture conditions by allowing for the presence of three different states in our simulations: empty, cell of type A or cell of type B. The rest of the simulation is carried out in the same manner as previously described. We now have an additional parameter which determines the initial ratio between the two subpopulations. We introduced an interaction strength parameter which affects the motility rate of a cell and it is scaled according to the proportion of another cell. The interaction strength is now defined by; $M_i = m_i + r_j * I_{ij}$ where subscripts dictate the cell, m is the base motility without interaction, M is the final motility rate of a cell, r is the current proportion and I_{ij} is the interaction strength on i received by j. We have chosen this regime as it is a simple method of introducing interactions and r_j represents the proportions of a cell and the neighborhood which this is calculated in our current implementation includes the entire simulation space. These longer-range interactions can be more akin to chemokine signaling as opposed to shorter range contact induced interactions. Whilst there are a multitude of different regimes to implement, we are drawing our attention to distinguishing that a model with no interactions is insufficient in explaining the data. The interaction neighborhood can be modified by changing the neighbourhood size parameter (with a neighbour size of N representing a 3D Moore neighborhood of magnitude N). We have chosen a global neighbourhood size spanning the entire simulation space. A visualisation of a sample co-culture simulation can be seen in Figure S4.

Finally for further analysis, a 2D collapse of the 3D array is created. Here 2D array is created for each cell type used (in this case 2 cell types) with every x-y coordinate containing a 1 if there was a cell of this type in the z-direction of the 3D array.

QUANTIFICATION AND STATISTICAL ANALYSIS

Bayesian computation

We used an approximate Bayesian computation approach throughout this study. The general approach here was to create a large databank of simulations. Each realisation in our databank is initiated from a set of parameters, where our parameters of interested was drawn from a non-informative uniform random distribution. In order to reduce the size of the databank, an exploratory simulation was run to validate the limits of the prior to ensure it encompasses the posterior.

For a sample, either experimental or simulated measurement, we calculated the distance between this and each realisation in our databank using a weighted Euclidean distance (derived using a genetic algorithm). We set an acceptance threshold, any distances that fell below this value were accepted and were used to generate a posterior distribution.

In theory, for an infinitely large databank, as the threshold approaches zero the posterior distribution converges to a single value, this value is the true parameter value.

For mono-culture simulations we fit a truncated normal distribution to the posterior distribution using the packages fitdistrplus, truncnorm and extraDistr in R.

However, in practice this is computationally infeasible and as such we generated credible intervals. These are the 95% HDI of the posterior distribution, and we expect our true parameter value to lie in this interval, this calculated using the HDInterval package in R.

Weighted Euclidean distance of summary statistics was calculated using the general formula:

$$Distance = \sum_i^N w_i (ObsVal_i - SimVal_i)^2 \quad with \quad \sum_i^N w_i = 1$$

Validation of summary statistics

To validate the accuracy of a scheme of summary statistics in recovering invasion parameters, a set of sample simulations are run with pre-defined motility and proliferation rates, and these are compared to a dataset of random simulations. Summary statistics are calculated for the sample simulations as well as each realisation in a dataset and weighted Euclidean distance between the sample and each simulation in the dataset is calculated for a scheme of summary statistics, such as a combination of the diffusion and wave front coefficients.

Cell Reports, Volume *40*

Supplemental information

Quantification of spatial subclonal interactions

enhancing the invasive

phenotype of pediatric glioma

Haider Tari, Ketty Kessler, Nick Trahearn, Benjamin Werner, Maria Vinci, Chris Jones, and Andrea Sottoriva

Supplementary information

Origin Tumour	Condition	Doubling time (days)
SU-DIPG-VI	Bulk tumour	3.4
SU-DIPG-VI	VI-D10	4.2
SU-DIPG-VI	VI-E6	4.6
SU-DIPG-VI	50% VI-D10 + 50% VI-E6	4.4
SU-DIPG-VI	75% VI-D10 + 25% VI-E6	4.2
SU-DIPG-VI	25% VI-D10 + 75% VI-E6	4.3
HSJD-DIPG-007	Bulk tumour	2.2
HSJD-DIPG-007	007-F8	2.1
HSJD-DIPG-007	007-F10	1.9
HSJD-DIPG-007	50% 007-F8 + 50% 007-F10	2.0
HSJD-DIPG-007	75% 007-F8 + 25% 007-F10	2.1
HSJD-DIPG-007	90% 007-F8 + 10% 007-F10	2.1

Supplementary Table 1: Doubling time for SU-DIPG-VI and HSJD-DIPG-007 derived subclones in mono-culture and various co-culture. Doubling times derived from 2D in vitro growth assays, are not directly comparable to growth under invasion assays but highlight little effect of co-culture conditions on growth rates observed. SU-DIPG-VI clones and bulk tumour line growth rate (n=3). HSJD-DIPG-007 clones and bulk tumour line growth rate (n=1).
(Related to cell doubling times in STAR Methods)

Experiment ID	Condition	Number of images				Comment
		Day 0	Day 1	Day 2	Day 3	
1	100% VI-E6	3	3	3	3	In this assay VI-E6 was labelled with Venus
1	90% VI-E6 + 10% VI-D10	3	3	3	3	
1	70% VI-E6 + 30% VI-D10	7	6	6	9	
1	50% VI-E6 + 50% VI-D10	9	10	10	11	
1	40% VI-E6 + 60% VI-D10	4	8	8	8	
1	20% VI-E6 + 80% VI-D10	6	8	7	8	
2	100% VI-D10	4	4	4	4	In this assay VI-D10 was labelled with Venus
2	10% VI-E6 + 90% VI-D10	3	3	3	3	
2	50% VI-E6 + 50% VI-D10	3	2	2	2	
2	90% VI-E6 + 10% VI-D10	5	5	4	4	
3	100% VI-E6	3	2	2	1	In this assay VI-E6 was labelled with Venus
3	90% VI-E6 + 10% VI-D10	5	6	6	7	
3	50% VI-E6 + 50% VI-D10	5	6	6	6	
3	10% VI-E6 + 90% VI-D10	2	2	3	4	
4	100% 007-F8	4	4	4	4	In this assay 007-F8 was labelled with mCherry and 007-F10 was labelled with Venus
4	90% 007-F8 + 10% 007-F10	3	3	3	3	
4	75% 007-F8 + 25% 007-F10	6	6	6	6	
4	50% 007-F8 + 50% 007-F10	4	4	4	4	
4	100% 007-F10	4	4	4	4	

Supplementary Table 2: Summary of the number of timepoints taken per condition for invasion assays between SU-DIPG-VI and HSJD-DIPG-007 derived subclones. There are variations between number of images per condition between timepoints. This is due to out of focus images being left out from analysis. For SU-DIPG-VI the mCherry labelling was not detectably expressed. This is a known issue with RFP-like labelling and as such for clones from this cell line only the green channel was used to track a subpopulation. In all assays both subclones were labelled with either mCherry (red) or Venus (green)
Experiment ID 1 = Counts of images for assays where VI-E6 is labelled with Venus and tracked (Assay 1)**. Experiment ID 2 =** Counts of images for assays where VI-D10 is labelled with Venus and tracked. **Experiment ID 3 =** Counts of images for assays where VI-E6 is labelled with Venus and tracked (Assay 2). **Experiment ID 4 =** Counts of images for assays where 007-F8 is labelled with Venus and 007-F10 is labelled with mCherry.
(Related to experimental timepoints for Figures 4, 5 and 6 and Supplementary Figures 1, 2)

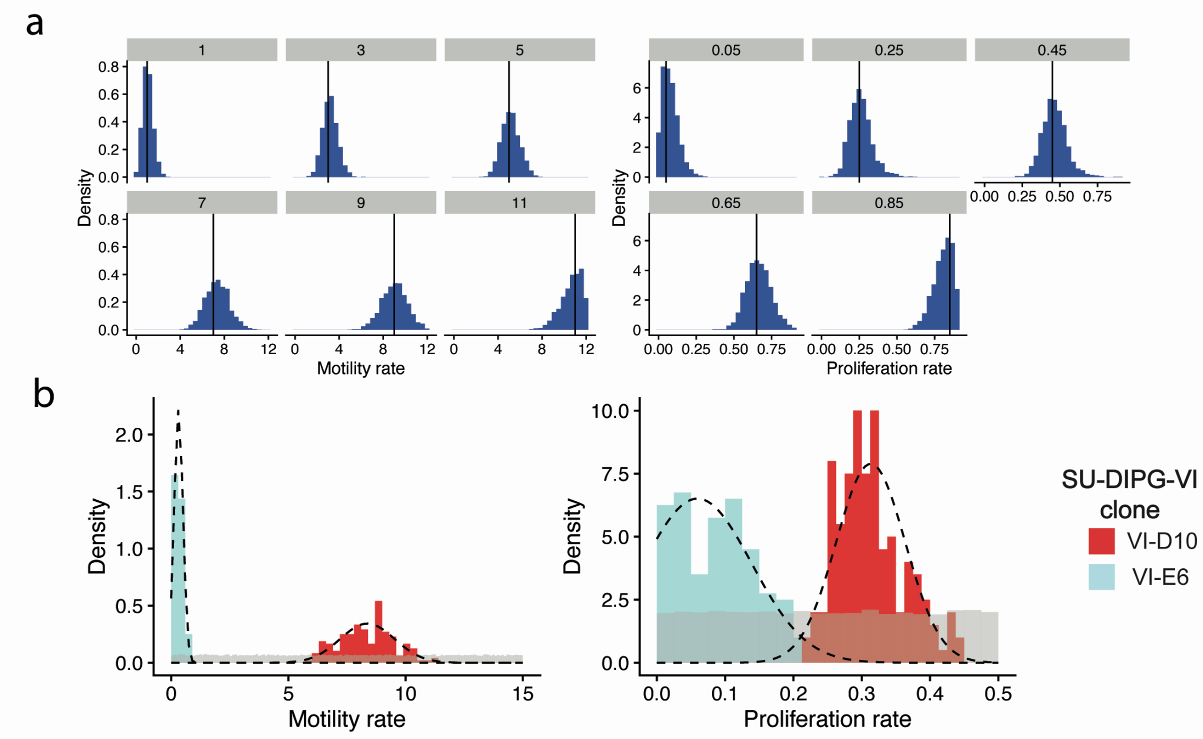

Supplementary figure 1: Recovery of invasion rates for SU-DIPG-VI clones VI-E6 and VI-D10 using area invaded with density of invaded area as summary statistics. a, Simulated recovery. **b,** Experimental recovery.

(Related to Figure 4)

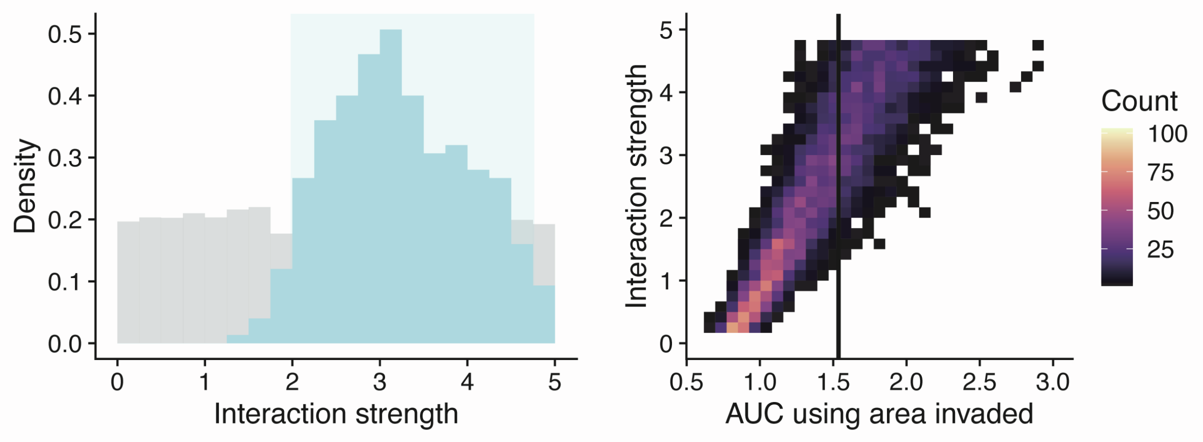

Supplementary figure 2: Additional replicate validates the experimental recovery of a positive interaction observed for SU-DIPG-VI clone VI-E6.

(Related to Figure 5)

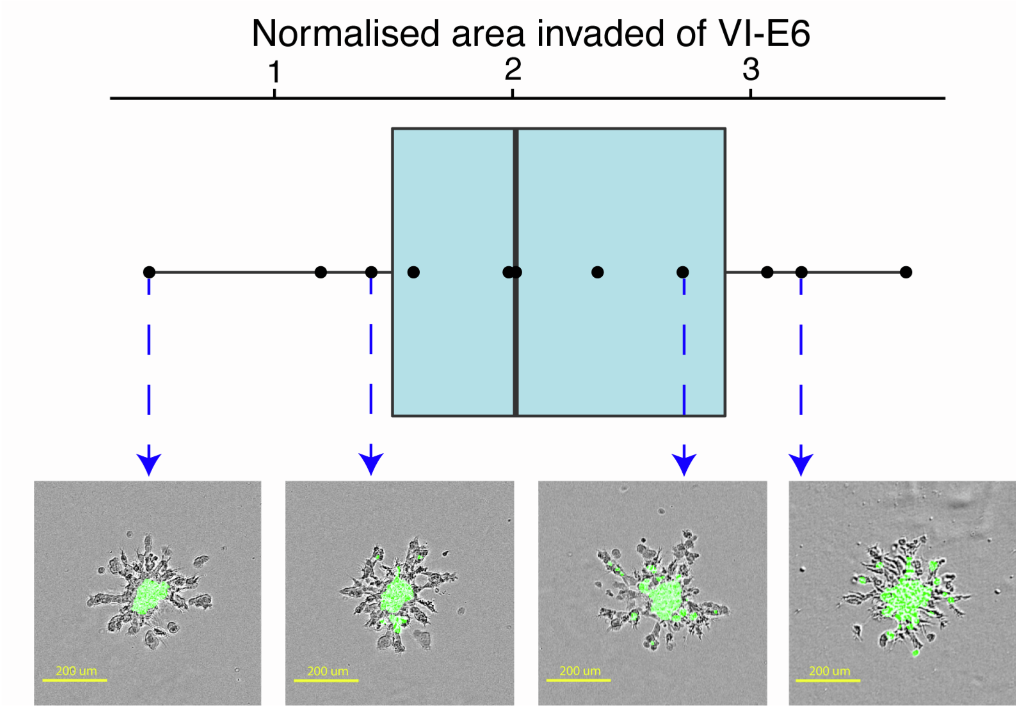

Supplementary figure 3: The interplay between spatial competition and positive interactions leads to variable outcomes in the invasive phenotype observed. Using VI-E6 (labelled in green) in a 50%-50% co-culture with VI-D10 as an illustrative example. Here the normalised area invaded of the green channel is measured**.** An overlay of the phase channel (showing all cells) with the green channel (showing only VI-E6 cells) demonstrates there is stochasticity in the interplay between interactions and spatial competition. In some replicates the spatial competition is able to prevent VI-E6 from invading, whilst in others VI-E6 appears free to invade in all directions (with minimal spatial restrictions). (Scale bars 200um).

(Related to Figure 5)

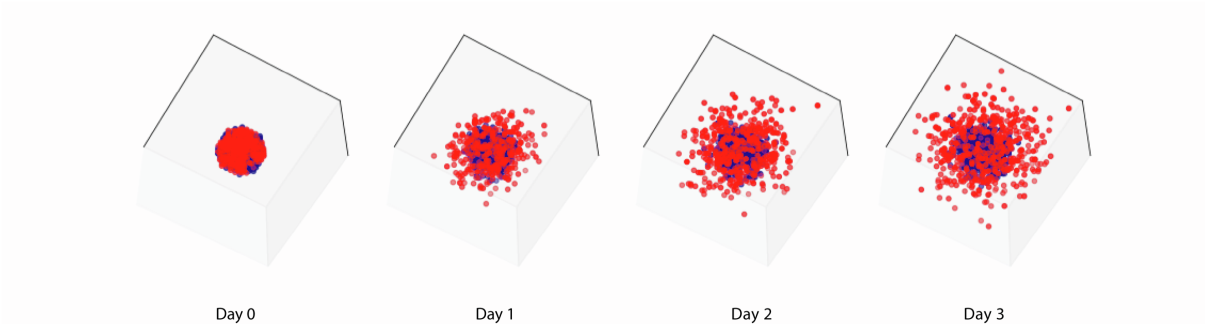

Supplementary figure 4: 3D visualisation of output from *in-silico* model of co-culture invasion. Two distinct cells are presented with identical proliferation rates (0.2 d/day) and differential motility rates. The population in red has a higher motility rate (10 x/day) than the blue population (1 x/day). **(Related to agent-based cellular automata in STAR Methods)**
